# Elevated UHRF1 predicts unfavorable clinical outcomes in adult T-cell leukemia/lymphoma: Clinicopathological analysis and preliminary in vitro observations

**DOI:** 10.1371/journal.pone.0362674

**Published:** 2026-11-11

**Authors:** Ying Lin, Rong-Dong Zhang, Xin-Yu Xu, Huang-zhou Jiang, Ren-Li Chen, Jian-Zhen Shen

**Affiliations:** 1 Department of Hematology, Fujian Institute of Hematology, Fujian Provincial Key Laboratory on Hematology, Fujian Medical University Union Hospital, Fuzhou, China; 2 Department of Hematology, Ningde Municipal Hospital, Ningde, China; 3 Department of Traditional Chinese Medicine, Ningde Municipal Hospital, Ningde, China; Duke University Medical Center: Duke University Hospital, UNITED STATES OF AMERICA

## Abstract

**Background:**

UHRF1 has been established as a susceptibility gene in multiple cancer types; however, its functional role in the pathogenesis of adult T-cell leukemia/lymphoma (ATLL) remains poorly defined. This study aimed to investigate UHRF1 expression in ATLL and assess its clinical and biological significance.

**Methods:**

Immunohistochemistry was performed to evaluate UHRF1 protein expression in tumor tissues from 72 ATLL patients and 30 reactive lymphadenopathy controls. Univariate Cox’s proportional hazard regression model was used to screen the factors affecting OS and PFS, and multivariate Cox’s proportional hazard regression model was used to screen independent prognostic factors in ATLL. UHRF1 expression in the HuT-102 ATLL cell line and MT-4 cell line was evaluated by Western blotting and quantitative PCR, and shRNA-mediated knockdown was used to assess proliferation and apoptosis.

**Results:**

UHRF1 protein was highly expressed in primary ATLL samples (P < 0.001, median 26.562 vs. 1.306), and the percentage of positive pixels (%Area) significantly higher than that in the control group (P < 0.001, median 9.914 vs. 0.574). Multivariate Cox regression analysis demonstrated that UHRF1 expression remained an independent prognostic predictor after adjustment for clinicopathological characteristics. For overall survival (OS): P = 0.026, HR = 2.397, 95%CI: 1.108–5.184; for progression-free survival (PFS): P = 0.042, HR = 1.959, 95%CI: 1.025–3.744. Mechanistically, UHRF1 knockdown significantly suppressed proliferation and induced apoptosis in ATLL cell lines (HuT-102 cells and MT-4 cell). Co-immunoprecipitation detected no stable interaction between UHRF1 and either AMPK or the PP2A catalytic subunit PPP2CA, and UHRF1 knockdown did not significantly alter LKB1, PPP2R4, or the p-AMPK (Thr172)/AMPK ratio; whether UHRF1 intersects with AMPK signaling remains to be determined. These findings identify UHRF1 as an independent adverse prognostic indicator in ATLL and provide preliminary biological evidence, though the underlying mechanisms require further validation in larger cohorts and in vivo models.

## Introduction

Adult T-cell leukemia/lymphoma (ATLL) is a highly aggressive malignancy characterized by the clonal expansion of mature CD4^+^ T lymphocytes, driven by persistent infection with human T-cell leukemia virus type I (HTLV-1) [1]. Emerging evidence suggests that the interplay between HTLV-1 mediated oncogenesis and dysregulated epigenetic mechanisms plays a pivotal role in adult ATLL pathogenesis [2,3].

UHRF1 (ubiquitin-like with PHD and ring finger domains 1) is a multifunctional epigenetic regulator essential for the maintenance of DNA methylation, DNA damage response, and chromatin remodeling. Beyond its canonical roles in epigenetic inheritance, UHRF1 also contributes to embryonic development, cell migration, and tumorigenesis [4–6]. UHRF1 overexpression has been documented in multiple hematological malignancies. In B-cell acute lymphoblastic leukemia (B-ALL), UHRF1-mediated epigenetic reprogramming has been shown to promote glycolysis and disease progression [7]. Aside from its metabolic regulatory function in hematologic cancers, UHRF1 can also modulate core inflammatory oncogenic pathways to drive tumorigenesis in solid tissue contexts. In prostate cancer, UHRF1 has been linked to NF-κB signaling and tumor progression [8]. However, the functional role of UHRF1 in T-cell malignancies, particularly ATLL, remains poorly defined.

ATLL exhibits substantial clinical heterogeneity, and current prognostic stratification relies heavily on clinical staging with limited integration of molecular biomarkers. Given the established involvement of UHRF1 in epigenetic silencing and its documented oncogenic role in other hematologic neoplasms, we hypothesized that UHRF1 may be aberrantly expressed in ATLL and associated with aggressive disease features and adverse clinical outcomes. This study was therefore designed to (1) evaluate UHRF1 protein expression and determine its independent prognostic significance in a clinical ATLL cohort, and (2) perform preliminary in vitro assessments of UHRF1 expression and its phenotypic associations in an ATLL cell line to inform future mechanistic investigations.

## Materials and methods

### Participants

Seventy-two patients with ATLL, either previously diagnosed or newly diagnosed between January 2018 and January 2025 at Ningde Municipal Hospital and Fujian Medical University Union Hospital, were enrolled in this study. All diagnoses were confirmed by histopathological examination of lymph node or bone marrow biopsies, immunohistochemistry (IHC), and supplementary diagnostic assay (including flow cytometry, HTLV-1 serology, and/or proviral load quantification) and fulfilled both established domestic (Chinese) and international (e.g., WHO and Shimoyama) diagnostic criteria for ATLL. In addition, 30 patients with reactive lymphadenopathy were included in this study as control (control group).

This study was approved by the Medical Research Ethics Committee of Ningde Municipal Hospital Affiliated to Ningde Normal University (approval number: NSYKYLL-2024-132). All procedures involving human participants were conducted in accordance with the ethical standards of the institutional and/or national research committee and with the 1964 Helsinki Declaration and its later amendments or comparable ethical standards.

### Data and samples collection

Formalin-fixed, paraffin-embedded (FFPE) tissue samples were collected from patients pathologically diagnosed with ATLL. Clinical and laboratory parameters were retrospectively retrieved from electronic medical records as of March 18, 2025, including Ann Arbor disease stage, HTLV-1 proviral load, complete blood count (CBC) indices, serum lactate dehydrogenase (LDH), C-reactive protein (CRP), erythrocyte sedimentation rate (ESR), serum β_2_-microglobulin, and Ki-67 proliferation index. This study was approved by the Medical Research Ethics Committee, and the requirement for informed consent was waived due to the retrospective nature of the analysis. All data were fully de-identified, and no personally identifiable information of subjects was accessible.

### Immunohistochemistry (IHC) and scoring

UHRF1 protein expression was evaluated by immunohistochemical staining performed on 4-μm FFPE tissue sections. Following deparaffinization and rehydration, antigen retrieval was conducted using citrate buffer (pH 6.0) under high pressure. Endogenous peroxidase activity was blocked with 3% hydrogen peroxide, and nonspecific binding was prevented by incubation with 5% bovine serum albumin (BSA). Sections were then incubated overnight at 4°C with a primary antibody (rabbit anti-human UHRF1 Polyclonal antibody, Proteintech, Catalog number: 21402-1-AP, 1:100 dilution). After washing, sections were incubated with horseradish peroxidase (HRP)-conjugated secondary antibody (Anti-rabbit IgG, CST, Catalog number: #7074, 1:10000 dilution) followed by visualization using 3,3’-diaminobenzidine (DAB) chromogen. Hematoxylin was used for counterstaining. Negative control sections were processed by omitting the primary antibody.

UHRF1 expression was scored independently by two experienced pathologists blinded to clinical outcomes. The DAB chromogen signal was quantified using ImageJ software (version 1.53; National Institutes of Health, USA). After color deconvolution to separate the DAB channel from the hematoxylin counterstain, a uniform intensity threshold was applied to exclude background signals. For each tissue section, three representative regions of interest (ROIs) were selected from tumor-dense areas by two independent pathologists, deliberately avoiding necrosis, hemorrhage, fibrous stroma, and non-tumoral inflammatory infiltrates. The following parameters were extracted for each region of interest (ROI): Area (total measured area, μm^2^), Mean (mean optical density of positive pixels), IntDen (integrated density, calculated as Area×Mean), %Area (percentage of positive pixels within the ROI), and RawIntDen (raw integrated density before optical density calibration). The IntDen served as the primary quantitative indicators of UHRF1 expression intensity and proportion, respectively. The final value for each patient was calculated as the mean of the three ROI measurements. Patients were stratified into high-expression and low-expression groups using the median IntDen as the cutoff.

### Cell culture

The human ATLL-derived cell line HuT-102 and MT-4 cell were obtained from Procell (Wuhan, China, catalog number: CL-0121 and CL‑0655, respectively). Cells were maintained in RPMI 1640 medium supplemented with 10% fetal bovine serum (FBS), 100 U/mL penicillin, and 100 μg/mL streptomycin, and cultured at 37°C in a humidified incubator with 5% CO_2_. Routine cell maintenance was carried out in T‑25 flasks, while T‑75 flasks were used for large‑scale cell expansion for subsequent experiments.

### Lentiviral shRNA transduction and stable knockdown

Short‑hairpin RNAs (shRNAs) targeting human *UHRF1* (Gene ID: 29128) and a scrambled negative control were cloned into the lentiviral vector GV493 (hU6‑MCS‑CBh‑gcGFP‑IRES‑puromycin; cloning sites: AgeI/EcoRI) by Gene JiKe (Shanghai, China). Three shRNA target sequences against UHRF1 were tested: 5′‑GCGCCCGCACCATCATCAAGT‑3′ (KD1, P25C1494), 5′‑GGACGAAGTCTTCAAGATTGA‑3′ (KD2, P25C1495), and 5′‑GCGACATGGCCTTCCACATCT‑3′ (KD3, P25C1496). The scrambled negative‑control shRNA (CON313) sequence was 5′‑TTCTCCGAACGTGTCACGT‑3′. Correct insertion of shRNA cassettes was verified by Sanger sequencing using the H1‑F primer (5′‑GGAAAGAATAGTAGACATAATAGC‑3′). Recombinant lentiviral particles were packaged with psPAX2 and pMD2.G helper plasmids. The lentiviral titers and volumes applied for cell transduction were: negative control (CON313), 1.7 × 10^9^ TU/mL, 1.18 µL; KD1 (LV‑UHRF1‑RNAi‑P25C1494), 6.4 × 10^8^ TU/mL, 3.13 µL; KD2 (LV‑UHRF1‑RNAi‑P25C1495), 1.9 × 10^9^ TU/mL, 1.05 µL; KD3 (LV‑UHRF1‑RNAi‑P25C1496), 1.1 × 10^9^ TU/mL, 1.82 µL. HuT‑102 and MT‑4 suspension cells were seeded at 2 × 10^5^ cells per well in 6‑well plates and incubated with lentiviral particles at an MOI of 10 in complete medium containing 8 μg/mL polybrene (Sigma‑Aldrich). After 24 h of transduction, medium was refreshed with complete RPMI 1640 medium. At 48 h post‑transduction, puromycin was supplemented at a final concentration of 2 µg/mL to select stably transduced polyclonal cell pools. Among the three shRNAs, KD2 showed the greatest UHRF1 knockdown efficiency and was therefore used for all subsequent functional experiments. Stable polyclonal cells were maintained and used for assays within passages 3‑6, and grouped as NC‑shRNA (NC) and UHRF1‑shRNA‑KD2 (KD2). Knockdown efficiency of UHRF1 was validated by Western blotting and qRT‑PCR.

### Quantitative real-time PCR (qRT-PCR)

Total RNA was extracted from HuT-102 cells and MT-4 cell using TRIzol reagent (Thermo Fisher Scientific) according to the manufacturer's instructions. RNA purity and concentration were assessed by NanoDrop spectrophotometry (A260/A280 ratio ~1.8–2.0). First-strand complementary DNA (cDNA) was synthesized from 1 μg of total RNA using the PrimeScript RT Reagent Kit (TaKaRa). qRT-PCR was performed on an ABI 7500 Fast Real-Time PCR System (Applied Biosystems) using SYBR Green Premix Ex Taq (TaKaRa). The thermocycling conditions consisted of an initial denaturation at 95°C for 30 seconds, followed by 40 cycles of 95°C for 5 seconds and 60°C for 34 seconds. Each sample was analyzed in triplicate, and relative mRNA expression levels were calculated using the 2^(-ΔΔCt) method, with GAPDH or β-actin serving as the internal reference gene. The primer sequences used in this study were UHRF1 forward primer: AGGTGGTCATGCTCAACTACA, reverse primer: CACGTTGGCGTAGAGTTCCC; GAPDH forward primer: TGACCACAGTCCATGCCATCAC, reverse primer: GCCTGCTTCACCACCTTCTTGA.

### Western blotting

Total protein was extracted from HuT-102 cells and MT-4 cell using radioimmunoprecipitation assay (RIPA) lysis buffer supplemented with phenylmethylsulfonyl fluoride (PMSF) and a protease/phosphatase inhibitor cocktail. Protein concentrations were quantified using the bicinchoninic acid (BCA) assay. Equal amounts of protein (20–40 μg per lane) were separated by 10% sodium dodecyl sulfate-polyacrylamide gel electrophoresis (SDS-PAGE) and transferred onto polyvinylidene difluoride (PVDF) membranes. After blocking with 5% non-fat milk or 5% BSA in Tris-buffered saline containing 0.1% Tween-20 (TBST) for 1 hour at room temperature, membranes were incubated overnight at 4°C with primary antibodies. Primary antibody information was as follows: rabbit polyclonal anti‑UHRF1 (Proteintech, Catalog number: 21402‑1‑AP, 1:2000 dilution), rabbit anti‑AMPK (Abways, Catalog number: CY5326, 1:1000 dilution), rabbit anti‑p‑AMPK (Thr172) (Cell Signaling Technology, Catalog number: #2535, 1:1000 dilution), rabbit anti‑LKB1 (HUABIO, Catalog number: HA500143, 1:1000 dilution), rabbit anti‑PPP2R4 (Proteintech, Catalog number: 10321‑1‑AP, 1:1000 dilution), mouse anti‑GAPDH (Santa Cruz Biotechnology, Catalog number: sc‑32233, 1:5000 dilution), and mouse anti‑β‑actin (Santa Cruz Biotechnology, Catalog number: sc‑69879, 1:8000 dilution). Following three washes with TBST, membranes were incubated with HRP-conjugated secondary antibodies for 1 hour at room temperature: HRP‑linked anti‑rabbit IgG (Cell Signaling Technology, Catalog number: #7074, 1:10000 dilution) and HRP‑linked anti‑mouse IgG (Cell Signaling Technology, Catalog number: #7076, 1:10000 dilution).

Protein bands were visualized using an enhanced chemiluminescence (ECL) detection system, and densitometric analysis was performed using ImageJ software (National Institutes of Health, USA). Original uncropped blot images are provided in S1 Raw Images.

### Cell proliferation assay

Cell proliferation was assessed using the Cell Counting Kit-8 (CCK-8; Dojindo, Japan) assay according to the manufacturer's instructions. Briefly, HuT-102 cells and MT-4 cell were seeded into 96-well plates at a density of 3 × 10^3^ cells per well after transfection. At designated time points (0, 1, 2, 3, 4, 5, and 6 days), 10 μL of CCK-8 solution was added to each well, and cells were incubated for an additional 2 hours at 37°C. The absorbance at 450 nm was measured using a microplate reader (Bio-Rad). Each experiment was performed with six replicate wells and repeated independently at least three times.

### Apoptosis assay

Apoptosis was quantified using an Annexin V-fluorescein isothiocyanate (FITC)/propidium iodide (PI) double-staining apoptosis detection kit (BD Biosciences). At 48 hours post-transfection, HuT-102 cells and MT-4 cell were harvested, washed twice with ice-cold phosphate-buffered saline (PBS), and resuspended in 1× binding buffer at a concentration of 1 × 10⁶ cells/mL. Subsequently, 5 μL of Annexin V-FITC and 5 μL of PI were added to 100 μL of cell suspension, followed by incubation in the dark for 15 minutes at room temperature. After the addition of 400 μL of binding buffer, samples were immediately analyzed by flow cytometry (BD FACSCanto II). Data were analyzed using FlowJo software (Tree Star, USA). Cells positive for Annexin V-FITC and negative for PI were identified as early apoptotic cells, whereas double-positive cells were considered late apoptotic or necrotic cells.

### Co-immunoprecipitation (Co-IP)

Two independent co-immunoprecipitation experiments were performed to examine the physical interactions between UHRF1 and AMPK-pathway components (AMPK and the PP2A catalytic subunit PPP2CA). HuT-102 cells were lysed in ice-cold Co-IP lysis buffer (20 mM Tris-HCl, pH 7.5, 150 mM NaCl, 1 mM EDTA, 1% NP-40, 10% glycerol, supplemented with protease and phosphatase inhibitors) for 30 minutes. Cell lysates were centrifuged at 12,000 × g for 15 minutes at 4°C, and the supernatants were precleared with Protein A/G agarose beads for 1 hour at 4°C. After preclearing, 500 μg of total protein was incubated overnight at 4°C with 2 μg of anti-UHRF1 antibody (Proteintech, Catalog number: 21402–1-AP; used at 1:200 for the AMPK-detection experiment and PPP2CA-detection experiment), or normal IgG as a negative control. Immune complexes were captured by adding 30 μL of Protein A/G agarose beads and rotating for 3 hours at 4°C. Beads were then washed four times with lysis buffer, and bound proteins were eluted by boiling in 2× SDS loading buffer for 5 minutes. For each experiment, immunoprecipitates (1,500 μg total protein per IP) and input lysates (40 μg) were resolved on 4−15% gradient SDS-PAGE gels and developed using ECL with X-ray film. The immunoprecipitated samples were subjected to Western blotting analysis as described above to detect interacting proteins. For immunoblot detection of pulled‑down proteins, anti‑UHRF1 (Proteintech, Catalog number: 21402‑1‑AP, 1:2000 dilution), anti‑AMPK (Proteintech, Catalog number: 66536‑1‑Ig, 1:2000 dilution), and anti-PPP2CA (Proteintech, Catalog number: 67809-1-Ig, 1/2000 dilution) antibodies were used.

### Statistical analysis

Statistical analyses were performed using SPSS version 26.0 (SPSS Inc, Chicago, USA). Categorical variables were presented as frequency (percentage), and intergroup comparisons were conducted using Chi-square test or Fisher’s exact test. Continuous variables were displayed as mean±standard deviation (SD) or median with interquartile range (IQR) according to the distribution, and the intergroup comparisons were analyzed using Student’s t-test or Wilcoxon rank sum test. Overall survival (OS) and progression-free survival (PFS) were estimated using the Kaplan–Meier method, with differences between groups assessed by the log-rank test. Univariate Cox’s proportional hazard regression model was used to screen the factors affecting OS and PFS, and multivariate Cox’s proportional hazard regression model was used to screen independent prognostic factors in ATLL. A two-sided P-value <0.05 was considered statistically significant.

## Result

### Basic information and clinical presentation

A total of 72 patients with ATLL were enrolled in this study, and their baseline demographic, clinical, pathological and laboratory parameters were summarized in Table 1. The mean age of the patients was 57.5 ± 12.4 years, with 37 males (51.4%) and 35 females (48.6%). Fifty percent of patients had an Eastern Cooperative Oncology Group performance status (ECOG-PS) score of 0, 38.9% had ECOG-PS 1, and 11.1% had ECOG-PS 2. According to ATLL subtype classification, 31 (43.0%) patients had acute-type disease, 21 (29.2%) lymphoma-type, 17 (23.6%) chronic-type, and only 3 (4.2%) smoldering-type. B symptoms and bone marrow infiltration were present in 81.9% and 90.3% of the cohort, respectively. Baseline laboratory data (median, IQR) were as follows: LDH 522.0 (393.0–633.8) U/L, β2-MG 4.9 (3.1–5.9) mg/L, Ki-67 60.0% (25.0–70.0%), CRP 24.3 (4.8–51.5) mg/L, and HTLV-1 viral load 6.2*10^6 IU/mL (0.6–50.2 *10^6 IU/mL). The median UHRF1 expression was 26.6 (19.8–34.6).

**Table 1:** Baseline patient and disease characteristics.

Patient characteristics	Patients (N = 72)
Age (year), mean±SD	57.5 ± 12.4
Sex, n (%)	
Male	37 (51.4)
Female	35(48.6)
ECOG-PS, n (%)	
0	36(50.0)
1	28(38.9)
2	8(11.1)
ATLL subtype, n (%)	
Acute	31(43.0)
Lymphoma	21(29.2)
Chronic	17(23.6)
Smoldering	3(4.2)
B symptoms, n (%)	59(81.9)
Bone marrow invasion, n (%)	65(90.3)
LDH (U/L), median (IQR)	522.0 (393.0-633.8)
β2-MG (mg/L), median (IQR)	4.9 (3.1-5.9)
Ki-67(%), median (IQR)	60.0 (25.0-70.0)
CRP (mg/L), median (IQR)	24.3 (4.8-51.5)
HTLV-1 virus amount (10^6 IU/mL), median (IQR)	6.2 (0.6-50.2)
UHRF1 expression, median (IQR)	26.6 (19.8-34.6)
UHRF1 expression, n (%)	
Positive	36 (50.0)
Negative	36 (50.0)

### Immunohistochemical detection of UHRF1 protein expression in pathological specimens of ATLL patients

Immunohistochemistry was employed to assess UHRF1 protein expression levels in ATLL group and control group. The results were quantitatively analyzed using ImageJ to determine the integrated density values and positive expression rates. Based on the obtained data, both the optical density (P < 0.001, median 26.562 vs. 1.306) and the positive rate of UHRF1 protein expression (P < 0.001, median 9.914 vs. 0.574) were significantly higher in the ATLL group compared to the control group (Fig 1).

**Fig 1:**
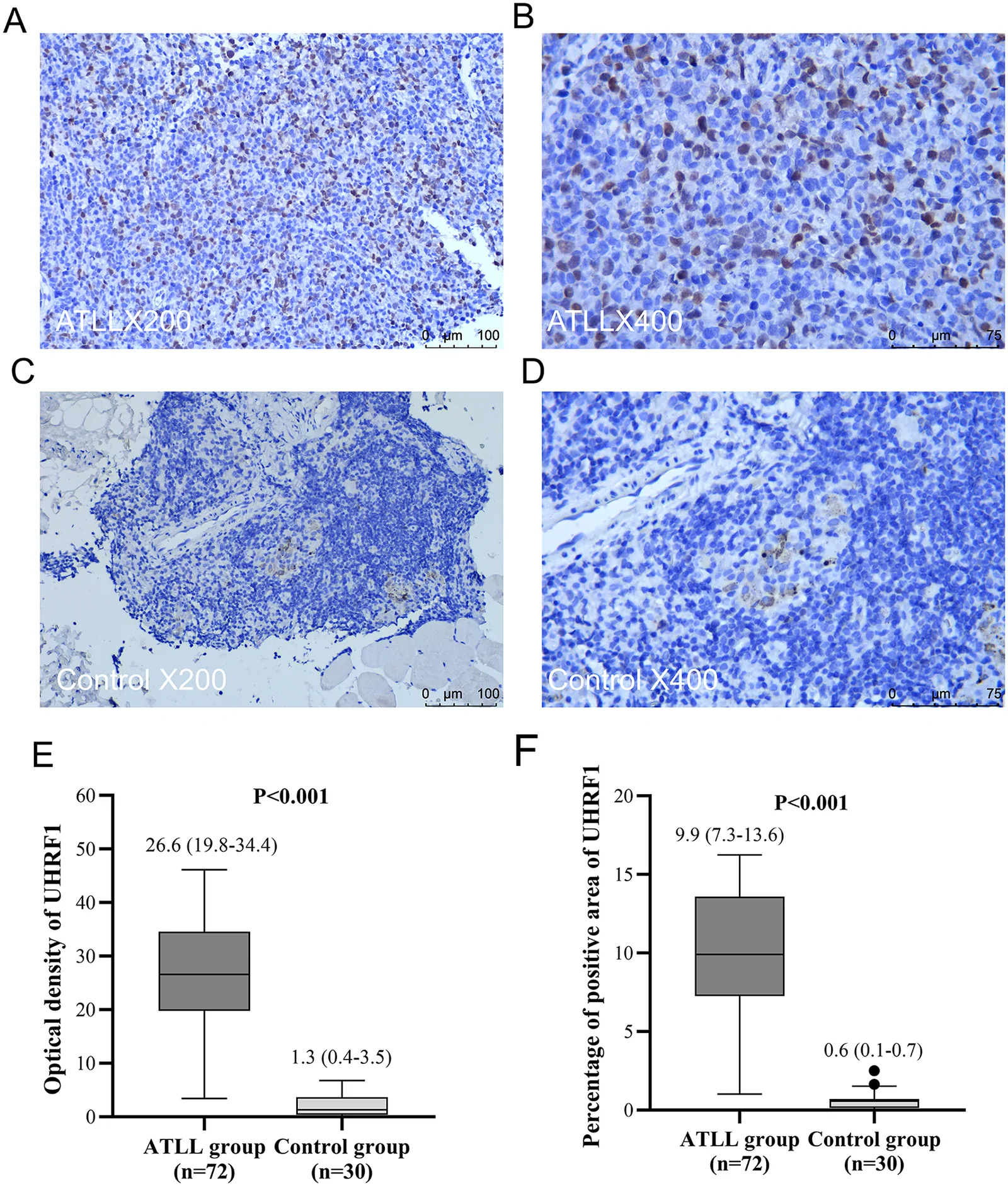
UHRF1 protein expression in pathological specimens from ATLL group and control group. **(A, B)** Representative immunohistochemical staining of UHRF1 in ATLL group at 200 × and 400× magnification, respectively. **(C, D)** Representative immunohistochemical staining of UHRF1 in control group at 200 × and 400× magnification, respectively. **(E, F)** Box‑plots showing the optical density (E) and positive area percentage (F) of UHRF1 immunohistochemical signals. Data are presented as median (interquartile range, IQR). The median optical density of UHRF1 was 26.6 (19.8‑34.4) in ATLL group (n = 72) versus 1.3 (0.4‑3.5) in control group (n = 30). The median positive area percentage was 9.9 (7.3‑13.6) for ATLL group and 0.6 (0.1‑0.7) for control group. The Wilcoxon rank sum test was applied for between‑group comparison. UHRF1 optical density and positive area percentage were both significantly higher in ATLL tissues than in control tissues (P < 0.001).

### The impact of UHRF1 protein expression on survival status

Kaplan-Meier survival analysis was performed to compare the OS and PFS between patients with positive and negative UHRF1 protein expression (Fig 2). Patients with high UHRF1 expression showed significantly worse PFS compared to those with low expression (P < 0.001, HR: 2.730; 95%CI: 1.563–4.769，Fig 2A). Similarly, the high UHRF1 expression patients have worse OS than in the low UHRF1 expression group (P < 0.001, HR: 3.416, 95%CI: 1.828–6.383, Fig 2B).

**Fig 2:**
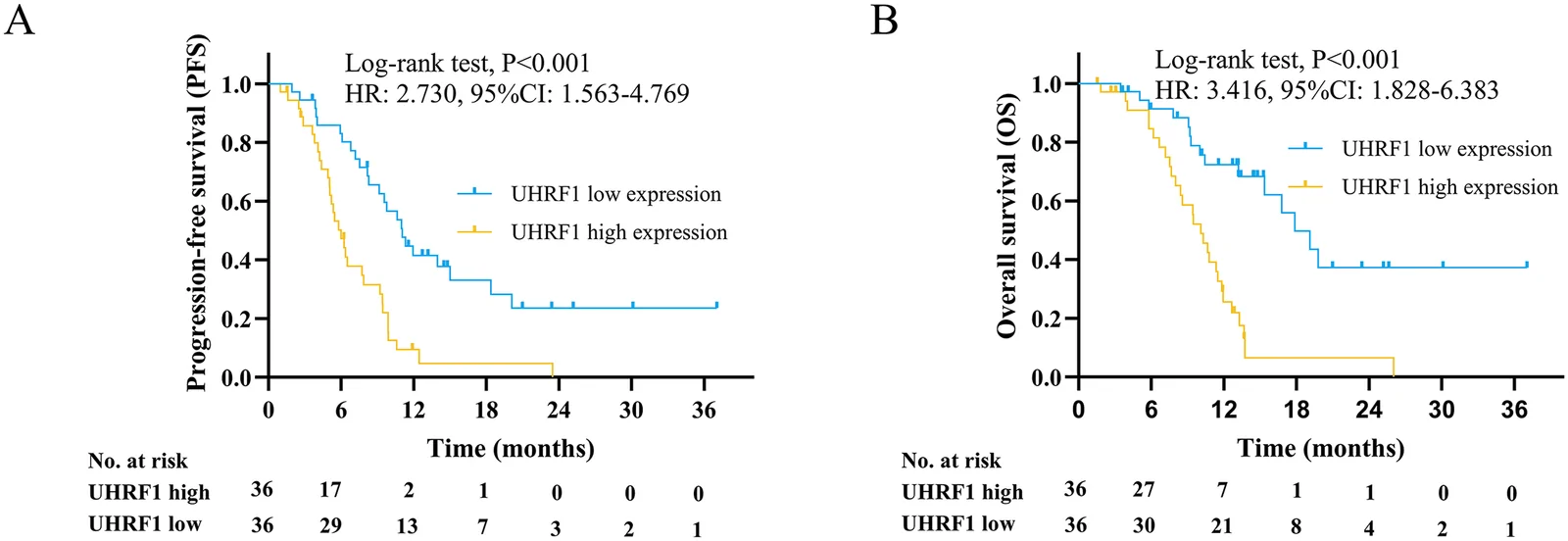
Kaplan-Meier survival analysis of PFS and OS stratified by UHRF1 expression in ATLL patients. **(A)** PFS was significantly shorter in patients with high UHRF1 expression versus low UHRF1 expression. **(B)** OS was markedly poorer in the high-UHRF1 group than in the low-UHRF1 group. Blue line: low UHRF1 expression; Yellow line: high UHRF1 expression. The log-rank test was applied for between‑group comparison.

### Correlation analysis of UHRF1 protein expression with clinical parameters in ATLL

As shown in Table 2, correlation analysis revealed that high expression of UHRF1 protein was significantly associated with several adverse clinical parameters in ATLL patients, including acute/lymphoma-type ATLL, B symptoms, bone marrow invasion, higher LDH, β2-MG, CRP, Ki-67 and HTLV-1 viral load. (all P < 0.05). In contrast, no significant associations were found between UHRF1 expression and patient age, sex, or ECOG-PS (all P > 0.05).

**Table 2:** UHRF1 expression and clinical characteristics of 72 ATLL patients.

Characteristics	UHRF1 Protein Expression		
	Low (n = 36)	High (n = 36)	P
Age (years), mean (SD)	58.2 ± 11.4	56.8 ± 13.4	0.631
Sex, n (%)			0.814
Male	18(50.0)	19(52.8)	
Female	18(50.0)	17(47.2)	
ECOG-PS, n (%)			0.602
0	20 (55.6)	16 (44.4)	
1	12 (33.3)	16 (44.4)	
2	4 (11.1)	4 (11.1)	
ATLL subtype, n (%)			0.002
Acute	13(36.1)	18(50.0)	
Lymphoma	6(16.7)	15(41.7)	
Chronic	14(38.9)	3(8.3)	
Smoldering	3(8.3)	0(0.0)	
B symptoms, n (%)			<0.001
Yes	23 (63.9)	36 (100.0)	
No	13 (36.1)	0 (0.0)	
Bone marrow invasion, n (%)			0.005
Yes	29(80.6)	36(100.0)	
No	7(19.4)	0(0.0)	
LDH (U/L), median (IQR)	482.0 (257.8-590.0)	578.5 (508.0-692.0)	0.004
β2-MG (mg/L), median (IQR)	3.5 (2.7-5.3)	5.1 (4.5-6.2)	0.004
CRP (mg/L), median (IQR)	5.2 (3.6-42.4)	37.2 (6.4-57.8)	0.011
Ki-67(%), median (IQR)	50.0 (20.0-70.0)	60.0 (50.0-70.0)	0.005
HTLV-1 virus amount (10^6 IU/mL), median (IQR)	3.3 (0.3-15.4)	8.8 (4.6-69.4)	0.005

### Analysis of factors influencing prognosis

The median duration of follow-up was 10.5 months (range: 1.5–37.0 months). During follow-up, 56 patients experienced disease progression, 43 patients died, and no patients were lost to follow‑up. Univariate and multivariate Cox’s proportional hazard regression model was performed to identify factors associated with PFS (Table 3). Univariate Cox regression was first performed to screen prognostic factors for PFS. Univariate analysis revealed that acute ATL, B symptoms, bone marrow invasion, elevated LDH, β2-MG, CRP, Ki-67 and high UHRF1 expression were significantly correlated with shortened PFS (all P < 0.05). All variables with statistical significance in univariate analysis were further enrolled into multivariate Cox proportional hazards regression model. After adjustment for confounding clinical and laboratory parameters, high Ki-67 expression (HR = 1.027, 95%CI: 1.000–1.055, P = 0.046) and high UHRF1 expression (HR = 1.959, 95%CI: 1.025–3.744, P = 0.042) remained independent adverse prognostic factors for unfavorable PFS in ATLL patients.

**Table 3:** Univariate and multivariate Cox’s proportional hazard regression model analyses of factors predicting PFS.

Items	Cox’s regression model			
	*P* value	HR	95% CI	
			Lower	Higher
**Univariate Cox’s regression**				
Age	0.806	0.997	0.976	1.019
Sex (male vs. female)	0.614	1.145	0.677	1.937
ATLL type (acute vs. lymphoma/chronic/smoldering vs. acute)	**<0.001**	0.369	0.214	0.636
ECOG-PS	0.263	0.798	0.537	1.185
B symptoms (yes vs. no)	**<0.001**	8.963	3.119	25.757
Bone marrow invasion (yes vs. no)	**0.006**	7.475	1.787	31.268
LDH	**<0.001**	1.004	1.002	1.005
*β*2-MG	**<0.001**	1.561	1.303	1.87
CRP	**<0.001**	1.017	1.008	1.027
Ki-67	**<0.001**	1.039	1.025	1.054
HTLV-1 virus amount	0.142	1	1	1.001
UHRF1 protein expression (high vs. low)	**<0.001**	2.981	1.712	5.192
**Multivariate Cox’s regression**				
ATLL type (acute vs. lymphoma/chronic/smoldering)	0.782	0.91	0.466	1.776
B symptoms (yes vs. no)	0.656	1.485	0.26	8.483
Bone marrow invasion (yes vs. no)	0.909	1.123	0.154	8.216
LDH	0.345	1.001	0.999	1.004
*β*2-MG	0.785	1.043	0.769	1.416
CRP	0.257	0.992	0.979	1.006
Ki-67	**0.046**	1.027	1	1.055
UHRF1 protein expression (high vs. low)	**0.042**	1.959	1.025	3.744

Univariate Cox regression for OS demonstrated that ATL subtype, B symptoms, bone marrow invasion, LDH, β2-MG, CRP, Ki-67, HTLV-1 viral load and UHRF1 expression were significantly associated with overall survival (all P < 0.05) (Table 4). Notably, the extremely high HR values of B symptoms and bone marrow invasion in univariate analysis were caused by severe imbalance of endpoint events between subgroups, and these two covariates were thus excluded from subsequent multivariate regression. All remaining statistically significant univariate predictors were entered into multivariate Cox analysis, which confirmed that elevated Ki-67 (HR = 1.033, 95%CI:1.002–1.065, P = 0.039) and high UHRF1 expression (HR = 2.397, 95%CI:1.108–5.184, P = 0.026) served as independent unfavorable prognostic markers for poor OS in ATLL patients.

**Table 4:** Univariate and multivariate Cox’s proportional hazard regression model analyses of factors predicting OS.

Items	Cox’s regression model			
	*P* value	HR	95% CI	
			Lower	Higher
**Univariate Cox’s regression**				
Age	0.904	1.002	0.977	1.026
Sex (male vs. female)	0.734	1.11	0.608	2.025
ATLL type (acute vs. lymphoma/chronic/smoldering)	**<0.001**	0.305	0.164	0.571
ECOG-PS	0.347	0.809	0.52	1.259
B symptoms (yes vs. no)	**0.005**	49.362	3.249	749.887*
Bone marrow invasion (yes vs. no)	**0.036**	35.186	1.252	988.513*
LDH	**<0.001**	1.004	1.002	1.006
*β*2-MG	**<0.001**	1.845	1.469	2.316
CRP	**<0.001**	1.025	1.014	1.036
Ki-67	**<0.001**	1.055	1.034	1.077
HTLV-1 virus amount	**0.048**	1	1	1.001
UHRF1 protein expression (high vs. low)	**<0.001**	3.846	1.982	7.462
**Multivariate Cox’s regression**				
ATLL type (acute vs. lymphoma/chronic/smoldering)	0.45	0.747	0.351	1.592
LDH	0.553	1.001	0.998	1.004
*β*2-MG	0.352	1.207	0.812	1.793
CRP	0.97	1	0.985	1.015
Ki-67	**0.039**	1.033	1.002	1.065
HTLV-1 virus amount	0.901	1	0.999	1.001
UHRF1 protein expression (high vs. low)	**0.026**	2.397	1.108	5.184

* The extremely high HR and 95%CI were attributed to severe imbalance of endpoint events across the two comparison groups, and hence these two covariates were not incorporated into the multivariate regression model.

Sensitivity multivariate Cox proportional‑hazards regression analysis was performed to explore prognostic factors for PFS and OS. After adjusting for ATL subtype, B symptoms, bone marrow invasion, and β2‑MG, high UHRF1 protein expression remained an independent adverse prognostic factor for PFS (HR = 1.866, 95%CI: 1.015‑3.428, P = 0.044, S1 Table) and OS (HR = 1.862, 95%CI: 1.028‑3.373, P = 0.040, S2 Table).

### The effect of UHRF1 gene silencing on the proliferation and apoptosis of ATLL cells

To explore the biological role of UHRF1 in ATLL, we first constructed three shRNA interference plasmids targeting UHRF1 and evaluated their knockdown efficiency in two ATLL cell lines (HuT-102 and MT-4). As shown in Fig 3, qPCR and Western blotting confirmed that all three constructs reduced UHRF1 expression at both mRNA and protein levels (all P < 0.05). The KD2 target site exhibited the highest knockdown efficiency and was therefore selected for subsequent functional experiments.

**Fig 3:**
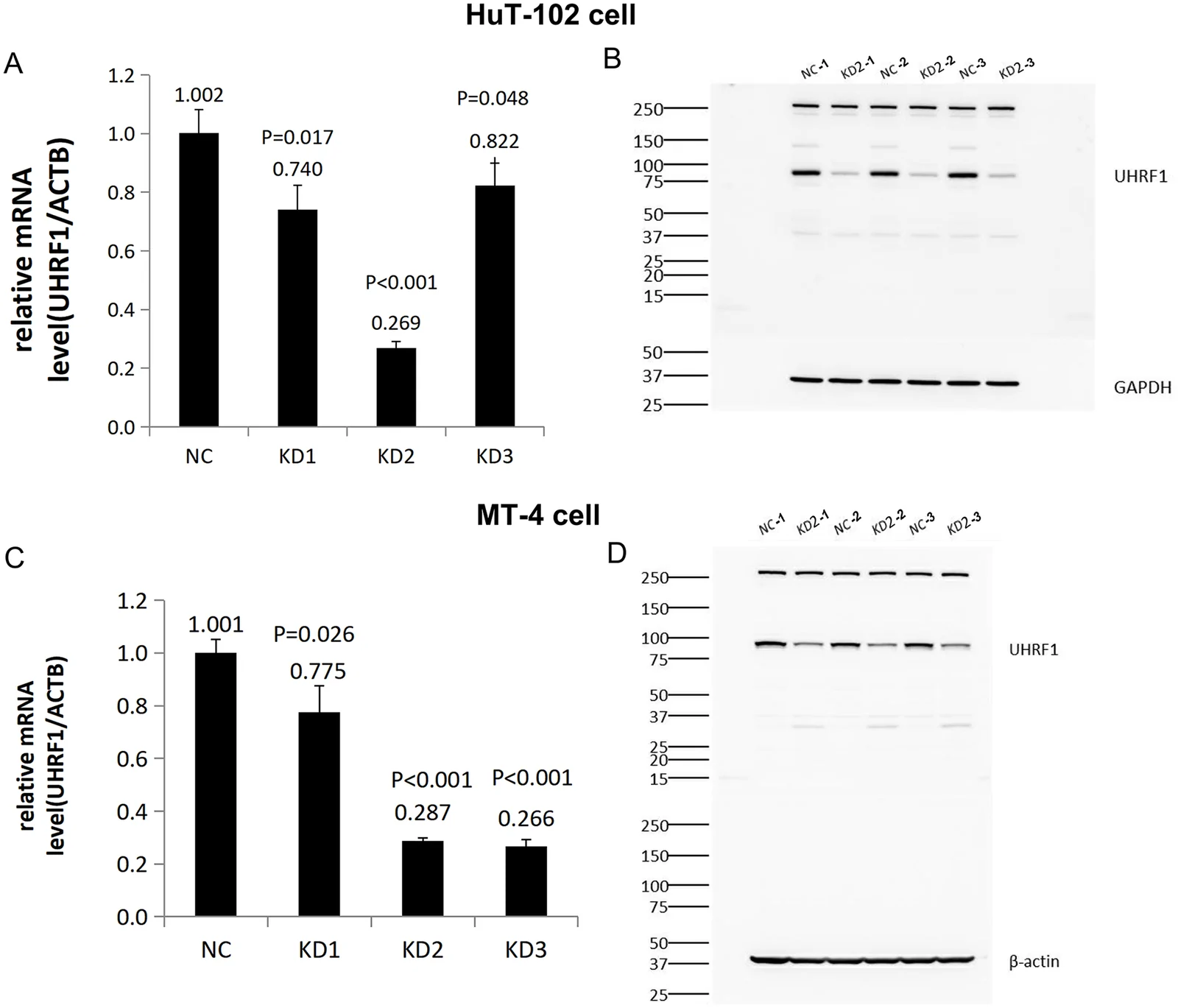
UHRF1 knockdown efficiency validation in ATLL HuT‑102 and MT‑4 cells. **(A)** qRT‑PCR detection of UHRF1 mRNA levels in HuT‑102 cells following UHRF1 knockdown with three independent shRNA constructs (KD1, KD2, KD3). UHRF1‑targeted shRNAs reduced UHRF1 mRNA abundance (0.740 ± 0.080, 0.269 ± 0.021, 0.822 ± 0.077 vs. 1.001 ± 0.080, P = 0.017，P < 0.001, and P = 0.048, respectively), and KD2 produced the most prominent silencing effect. UHRF1 mRNA values were normalized to ACTB. **(B)** Western‑blot analysis of UHRF1 protein in HuT‑102 cells after UHRF1 knockdown (KD2). The three NC lanes and the three KD2 lanes represent three independent biological replicates per group. GAPDH was used as the internal loading control. **(C)** qRT‑PCR analysis of UHRF1 mRNA expression in shRNA‑transduced MT‑4 cells. All three shRNA constructs down‑regulated UHRF1 mRNA (KD1, KD2, KD3: 0.775 ± 0.101, 0.287 ± 0.010, 0.266 ± 0.027 vs. 1.001 ± 0.050, P = 0.026，P < 0.001, and P < 0.001, respectively), with KD2 achieving the strongest knockdown efficiency. UHRF1 mRNA levels were normalized against ACTB. **(D)** Western‑blot verification of reduced UHRF1 protein abundance in UHRF1‑knockdown (KD2) MT‑4 cells. The three NC lanes and the three KD2 lanes represent three independent biological replicates per group. β‑actin served as the loading control. Data in panels A and C are presented as mean±standard deviation (SD) from three independent biological replicates (n = 3 per group). The Student’s t-test was applied for between‑group comparison. Based on the maximal silencing performance, the KD2 shRNA was selected for all subsequent functional experiments.

The effect of UHRF1 silencing on cell proliferation was assessed using the CCK-8 assay. Over a 5-day culture period, the absorbance (OD_450_) of the UHRF1-knockdown (KD2) group was significantly lower than that of the negative control (NC) group at all measured time points (Fig 4A/C, day2/3/4/5 all P < 0.05). When normalized to day 1, the fold change in cell viability was also markedly reduced in the KD2 group (Fig 4B/D, day2/3/4/5 all P < 0.05). Notably, UHRF1 knockdown induced a more striking proliferation arrest in MT‑4 cells, where KD2 cells barely expanded throughout the culture period. Statistical analysis confirmed that proliferation was significantly inhibited in KD2 cells compared with NC cells (P < 0.05), indicating that UHRF1 knockdown suppresses ATLL cell proliferation in vitro.

**Fig 4:**
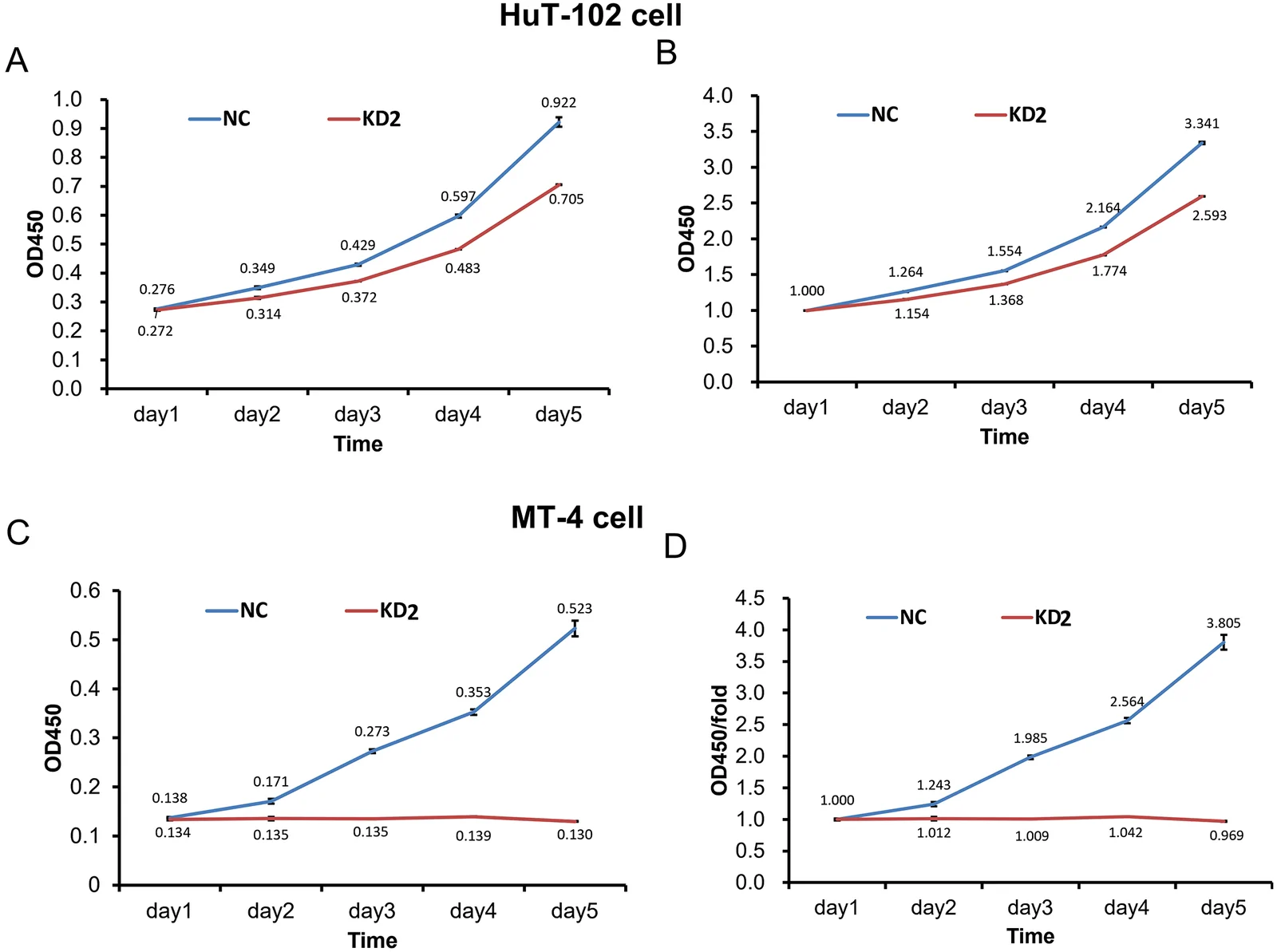
Proliferation profiles of UHRF1‑knockdown ATLL HuT‑102 and MT‑4 cells evaluated by CCK‑8 assay. **(A)** Time‑course OD_450_ absorbance values of UHRF1‑knockdown HuT‑102 cells across five consecutive days. OD_450_ absorbance is proportional to the number of viable cells. **(B)** OD_450_ fold‑change normalized to day 1 for UHRF1‑knockdown HuT‑102 cells. **(C)** Serial OD_450_ absorbance measurements of UHRF1‑knockdown MT‑4 cells during 5‑day culture. **(D)** OD_450_ fold‑change relative to day 1 for UHRF1‑knockdown MT‑4 cells. Data are presented as mean ± standard deviation (SD). Student's t‑test was used to compare NC and KD2 groups at each time point (n = 3 per group). In HuT‑102 cells, UHRF1 silencing significantly suppressed proliferation from day 2 onward (day 2: P = 0.001, day3, 4, and 5: all P < 0.001), with no significant difference at day 1 (P = 1.000). In MT‑4 cells, UHRF1 knockdown produced significant growth inhibition from day 2 onward (day 2: P = 0.001; day 3/4/5: P < 0.001), while day 1 showed no significant difference (P = 1.000).

Flow cytometry was performed to detect cell apoptosis and cell‑cycle changes after UHRF1 knockdown in HuT‑102 and MT‑4 cells (Fig 5). In HuT‑102 cells, UHRF1 silencing significantly increased early apoptotic (3.64 ± 0.09% vs. 2.48 ± 0.06%, P < 0.001) and late apoptotic cell proportions (2.19 ± 0.15% vs. 1.28 ± 0.06%, P < 0.001), and reduced viable cell percentage (74.85 ± 1.21% vs. 77.52 ± 0.38%, P = 0.022), while necrotic cell levels remained unchanged (P = 0.457). Cell‑cycle analysis showed that UHRF1 knockdown decreased G1 (34.3 ± 0.53 vs. 36.1 ± 0.32, P = 0.007) and G2/M phase proportions (9.0 ± 0.63 vs. 11.1 ± 0.96, P = 0.033) and increased S‑phase cell accumulation (56.7 ± 0.1 vs. 52.8 ± 0.97, P = 0.019).

**Fig 5:**
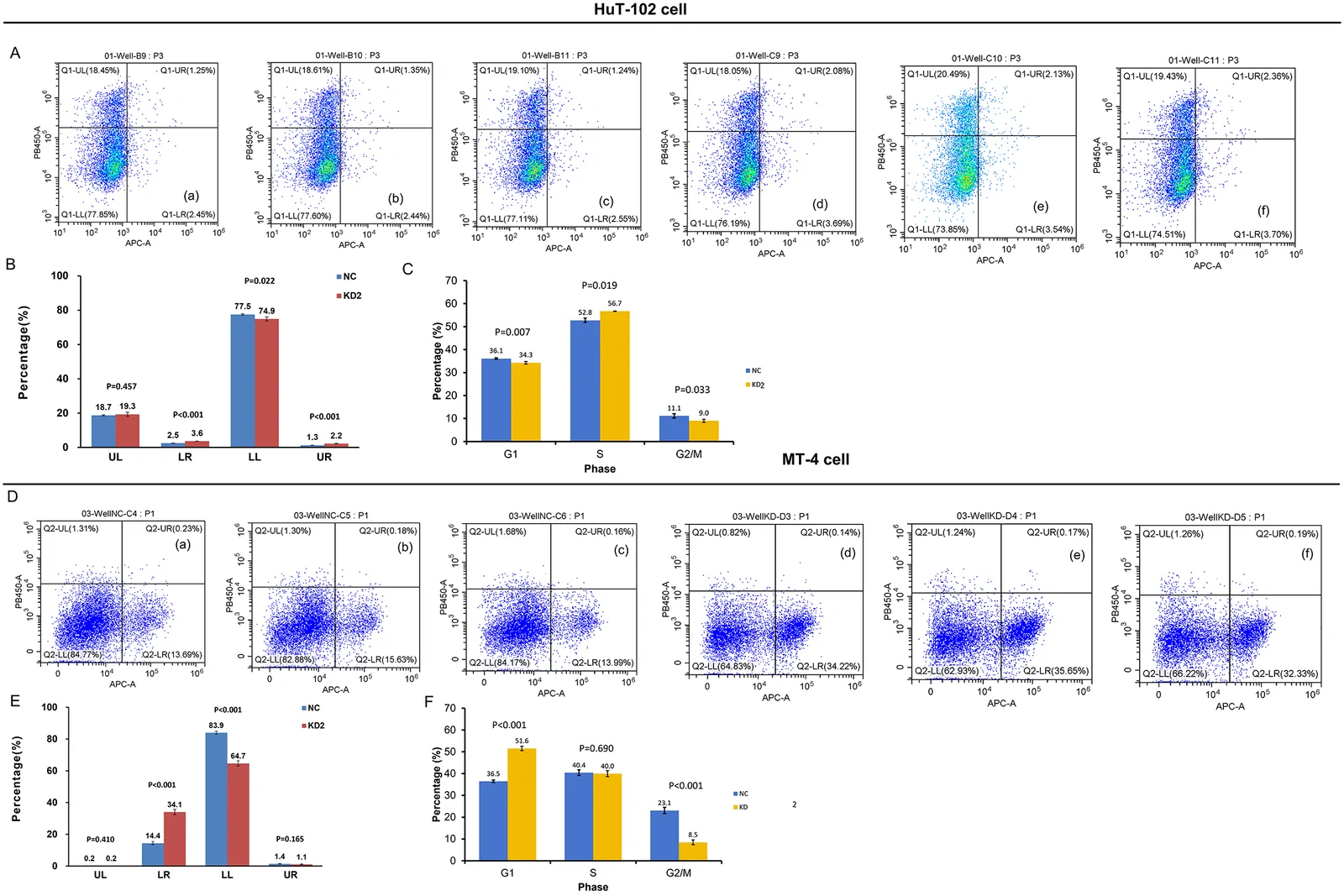
Effects of UHRF1 gene silencing on cell apoptosis and cell‑cycle distribution in ATLL HuT‑102 and MT‑4 cells. **(A)** Representative flow‑cytometry dot‑plots for apoptosis detection in HuT‑102 cells. Panels (a‑c) show biological replicates of the NC group; panels (d‑f) show biological replicates of the KD2 group. **(B)** Quantitative statistics of apoptotic cell populations in HuT‑102 cells. NC vs. KD2 UL: (18.72 ± 0.34% vs. 19.32 ± 1.22%, P = 0.457); LR (2.48 ± 0.06% vs. 3.64 ± 0.09%, P < 0.001); LL (77.52 ± 0.38% vs. 74.85 ± 1.21%, P = 0.022); UR: (1.28 ± 0.06% vs. 2.19 ± 0.15%, P < 0.001). **(C)** Quantitative analysis of cell‑cycle phase distribution (G1, S, G2/M) in HuT‑102 cells. UHRF1 knockdown significantly reduced the percentages of cells in the G1 (34.3 ± 0.53 vs. 36.1 ± 0.32, P = 0.007) and G2/M phases (9.0 ± 0.63 vs. 11.1 ± 0.96, P = 0.033), and induced a prominent increase in S‑phase cell proportion (56.7 ± 0.1 vs. 52.8 ± 0.97, P = 0.019). **(D)** Representative flow‑cytometry dot‑plots for apoptosis detection in MT‑4 cells. Panels (a‑c) represent biological replicates of the NC group; panels (d‑f) represent biological replicates of the KD2 group. **(E)** Quantitative analysis of apoptotic fractions in MT‑4 cells. NC vs. KD2 UL: (0.19 ± 0.04% vs. 0.17 ± 0.03%, P = 0.410); LR (14.44 ± 1.04% vs. 34.07 ± 1.67%, P < 0.001); LL (83.94 ± 0.97% vs. 64.66 ± 1.65%, P < 0.001); UR: (1.43 ± 0.22% vs. 1.11 ± 0.25%, P = 0.165). **(F)** Quantitative analysis of cell‑cycle phase distribution (G1, S, G2/M) in MT‑4 cells. UHRF1 knockdown markedly elevated the proportion of G1‑phase cells (51.6 ± 1.04 vs. 36.5 ± 0.56, P < 0.001), substantially decreased G2/M‑phase cell percentage (8.5 ± 1.09 vs. 23.1 ± 1.42, P < 0.001), and exerted no significant effect on the fraction of S‑phase cells (40.0 ± 1.38 vs. 40.4 ± 1.31, P = 0.690). Data in panels B, C, E, and F are presented as mean±standard deviation (SD) from three independent biological replicates (n = 3 per group). The Student’s t-test was used for between-group comparisons. UL: necrotic cells; LR: early‑apoptotic cells; LL: viable cells; UR: late‑apoptotic cells.

In MT‑4 cells, UHRF1 knockdown also induced obvious apoptosis, manifesting as a marked increase in early apoptotic cells (34.07 ± 1.67% vs. 14.44 ± 1.04%, P < 0.001) and a decrease in viable cells (64.66 ± 1.65% vs. 83.94 ± 0.97%, P < 0.001). No significant differences were observed in necrotic (P = 0.410) and late apoptotic cell proportions (P = 0.165). For cell cycle, UHRF1 silencing caused significant G1‑phase arrest (51.6 ± 1.04 vs. 36.5 ± 0.56, P < 0.001) and G2/M‑phase reduction (8.5 ± 1.09 vs. 23.1 ± 1.42, P < 0.001), whereas the S‑phase proportion was not altered (40.0 ± 1.38 vs. 40.4 ± 1.31, P = 0.690).

Collectively, these in vitro functional experiments demonstrate that UHRF1 plays a critical role in regulating the proliferation, apoptosis, and cell cycle progression of ATLL cells.

### Interaction between UHRF1 and the AMPK signaling pathway

To further explore the potential mechanism by which UHRF1 regulates ATLL cell proliferation, we investigated its functional interaction with the AMPK signaling pathway. Immunoblotting analysis confirmed that both total AMPK and phosphorylated AMPK (p-AMPK, Thr172) were detectable in HuT-102 (Fig 6).

**Fig 6:**
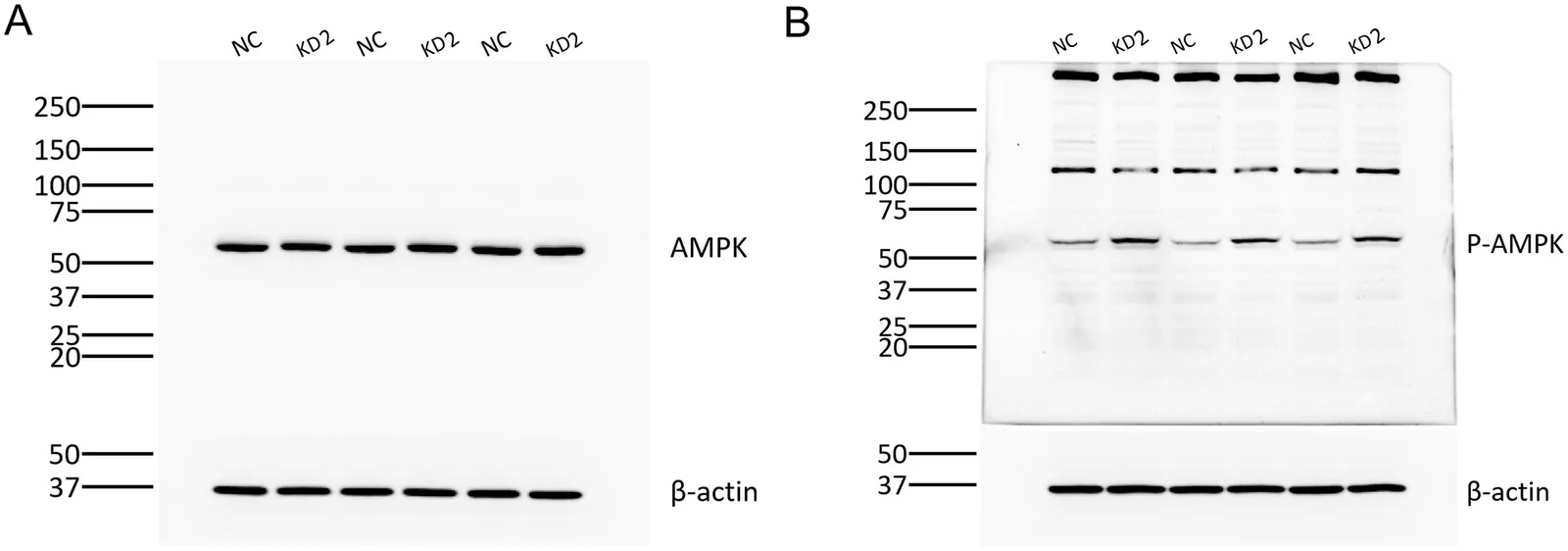
Detection of AMPK and phosphorylated AMPK (p-AMPK, Thr172) in HuT-102. **(A)** Total AMPK and **(B)** p-AMPK (Thr172) bands were detected in HuT-102, confirming that both total AMPK and its phosphorylated form are readily detectable in the cell.

Subsequently, immunofluorescence staining was performed to examine the subcellular localization of UHRF1 and PRKAA1 (AMPKα1). As shown in Fig 7, the green fluorescent signal (UHRF1) and red fluorescent signal (PRKAA1) exhibited partial overlap between UHRF1 and PRKAA1 fluorescence signals, suggesting that UHRF1 and PRKAA1 can reside within the same subcellular compartment. However, this finding alone cannot confirm their physical interaction.

**Fig 7:**
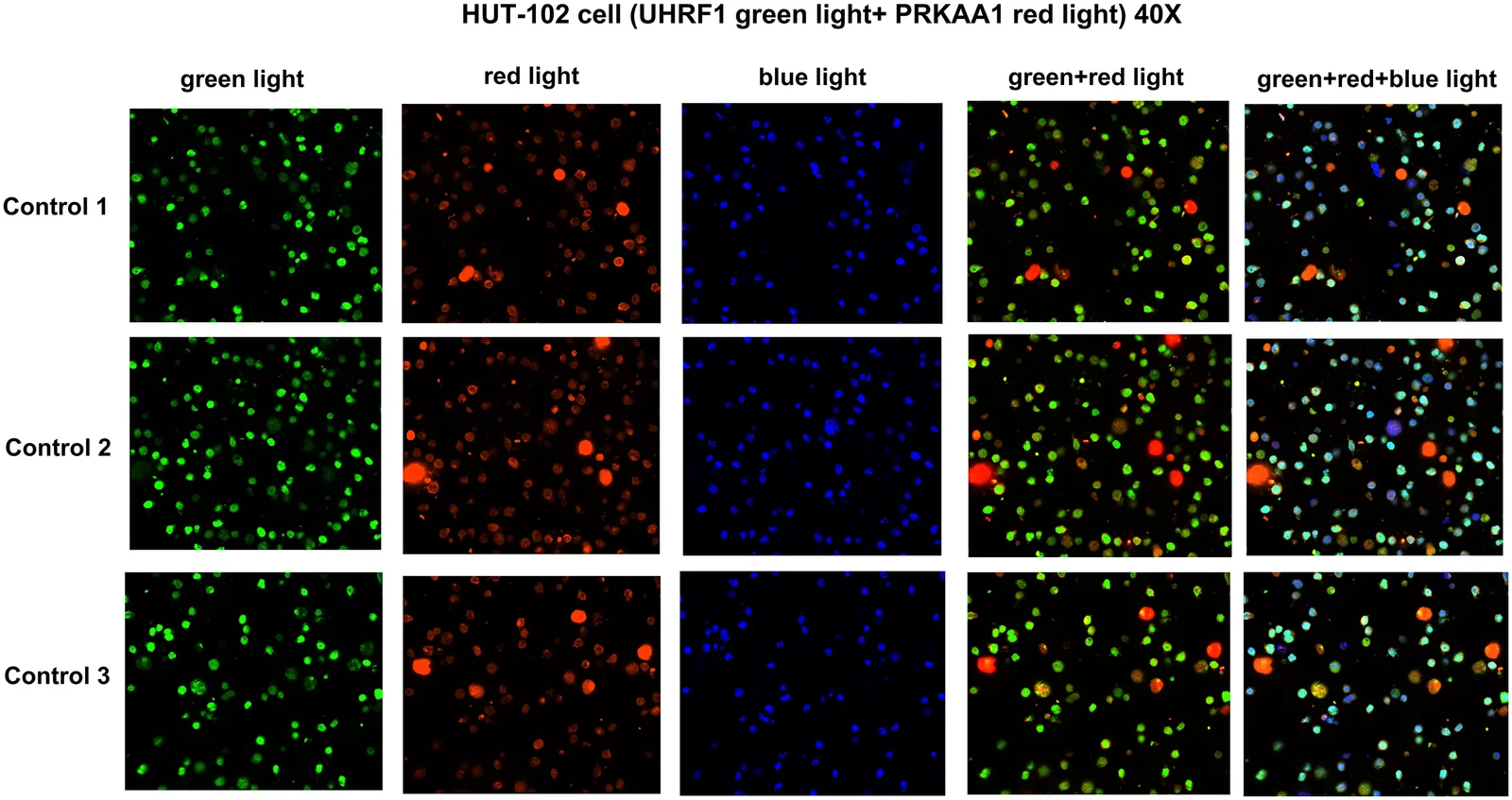
Subcellular localization of UHRF1 and AMPKα1 (PRKAA1) in ATLL cells by immunofluorescence. The immunofluorescence signals of UHRF1 and PRKAA1 (AMPK α1) in ATLL cells were visualized by fluorescence microscopy. Green fluorescent signal represents UHRF1, and red fluorescent signal represents PRKAA1 (AMPK Alpha 1). Cell nuclei were counter‑stained with DAPI (blue). Control 1‑3 represent three independent biological replicates.

### Exploration of the relationship between UHRF1 and the AMPK signaling axis

To determine whether UHRF1 directly interacts with AMPK pathway components, co-immunoprecipitation assays were performed in ATLL cells (Fig 8). Endogenous UHRF1 was efficiently immunoprecipitated (Fig 8A and 8B, upper panels). AMPK (~62 kDa) was readily detected in the input lysate but was not detectably co-immunoprecipitated with UHRF1 (Fig 8A, lower panel); likewise, the PP2A catalytic subunit PPP2CA (~36 kDa) was detected in the input but not in the UHRF1 immunoprecipitate (Fig 8B, lower panel). Because both AMPK and PPP2CA migrate outside the immunoglobulin heavy- (~50 kDa) and light-chain (~25 kDa) regions, these negative results are interpretable despite residual chain signals. These findings indicate that UHRF1 does not form a detectable stable complex with AMPK or PPP2CA under the tested conditions, suggesting that any functional crosstalk between UHRF1 and the AMPK axis is likely indirect, although weak or transient interactions cannot be excluded.

**Fig 8:**
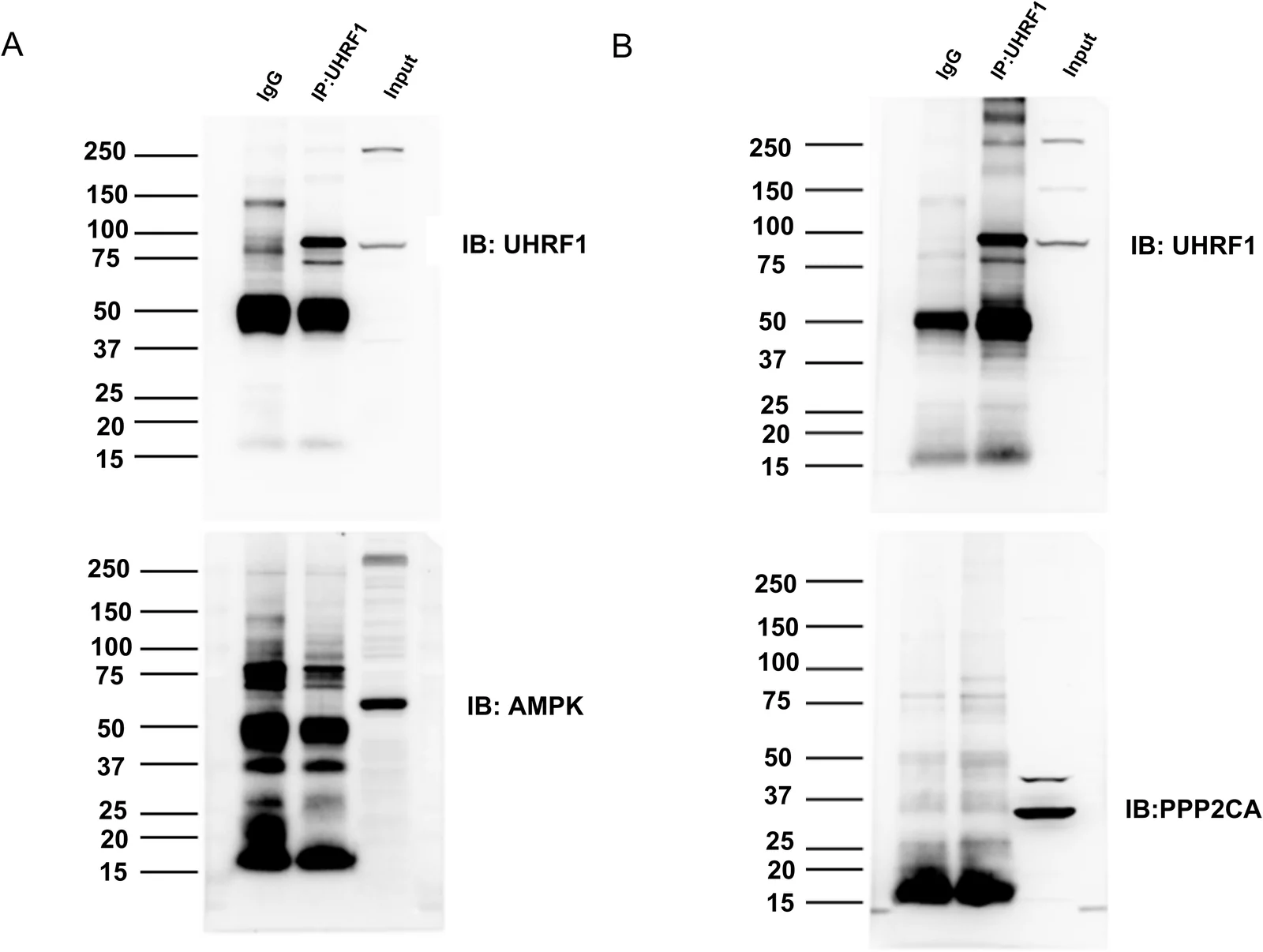
Co-immunoprecipitation analysis of interactions between UHRF1 and AMPK/PPP2CA. **(A, B)** Co‑immunoprecipitation experiments were performed to evaluate physical interactions between UHRF1 and its candidate binding partners. In Input lysates, distinct bands corresponding to endogenous UHRF1 (~91 kDa), AMPK (~63 kDa) (A), and PPP2CA(~33kDa) (B) were readily detected, confirming their expression in ATLL cells. A specific band for UHRF1 at ~91 kDa was observed in the IP‑UHRF1 group, verifying successful immunoprecipitation of UHRF1. Normal IgG was used as the negative control for immunoprecipitation. Neither AMPK nor PPP2CA was detectably co-immunoprecipitated with UHRF1 under the tested conditions. Bands at ~50 kDa and ~25 kDa in the IP lanes correspond to the immunoglobulin heavy and light chains, respectively.

We further examined the protein levels of the upstream AMPK regulators LKB1 and PPP2R4, as well as the p‑AMPK (Thr172)/total AMPK ratio, using three paired biological replicates of NC and UHRF1‑KD2 cells (Fig 9). No obvious changes in LKB1, PPP2R4, or the p‑AMPK/total AMPK ratio were observed upon UHRF1‑KD2. Of note, weak and variable band intensities precluded robust densitometric quantification and formal statistical testing. Taken together, although UHRF1 knockdown suppresses proliferation and induces apoptosis in ATLL cells, our current data do not demonstrate that these cellular phenotypes are mediated via altered AMPK phosphorylation; the mechanistic link between UHRF1 and the AMPK axis remains to be further defined.

**Fig 9:**
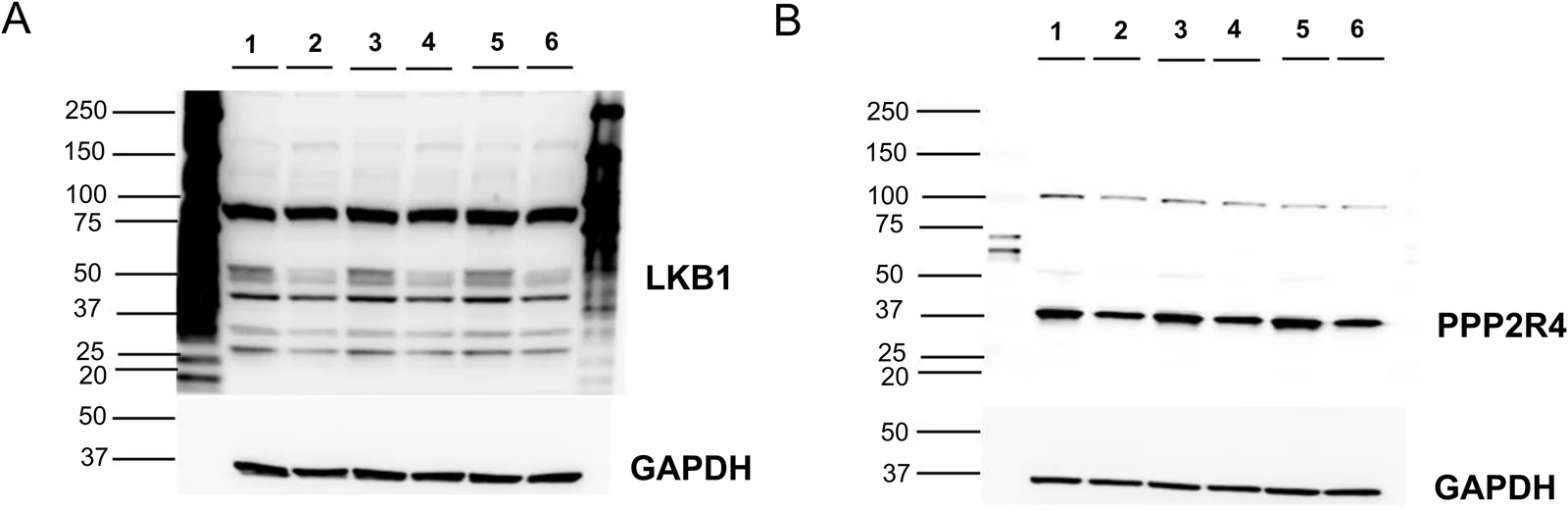
Western blot analysis of AMPK-pathway regulators following UHRF1 knockdown. **(A and B)** Western blot was performed to examine protein levels of LKB1 and the phosphatase subunit PPP2R4, key upstream regulators of AMPK. Lanes 1‑6 represent three paired biological replicates; lanes 1, 3, 5: NC group; lanes 2, 4, 6: UHRF1‑KD2 group. GAPDH was used as the loading control. Representative blots are shown; reliable densitometric quantification was not feasible due to weak and inconsistent band signals across biological replicates.

## Discussions

This study demonstrates UHRF1 as an independent adverse prognostic indicator in ATLL. In the retrospective cohort of 72 ATLL patients, high expression of UHRF1 protein was significantly associated with several adverse clinical parameters in ATLL patients, including acute/lymphoma-type ATL, B symptoms, bone marrow invasion, higher LDH, β2-MG, CRP, Ki-67 and HTLV-1 viral load. After adjustment for confounding clinical and laboratory parameters, high UHRF1 expression remained independent adverse prognostic factors for unfavorable PFS and OS in ATLL patients. In parallel, in vitro experiments demonstrated that UHRF1 knockdown significantly suppressed proliferation and induced apoptosis in ATLL cell lines (HuT-102 cells and MT-4 cell). In contrast, co-immunoprecipitation and immunoblot analyses did not establish a direct or clearly defined regulatory relationship between UHRF1 and the AMPK axis under the tested conditions. These findings identify UHRF1 as an independent adverse prognostic indicator in ATLL and provide preliminary biological evidence.

UHRF1 is a multifunctional nuclear protein that functions as a key component of a large macromolecular complex involved in the maintenance of epigenetic inheritance during DNA replication. The correlation between UHRF1 overexpression and aggressive clinicopathological features aligns with its established oncogenic role in other hematologic and solid malignancies [7–14]. A study in cervical cancer have shown that anti-UHRF1 antibodies can specifically and effectively label malignant cells [15]. Moreover, UHRF1 has been identified as a potential biomarker for pancreatic cancer, exhibiting significantly higher expression in pancreatic tumor tissues than in those from chronic pancreatitis or healthy pancreas [16]. Furthermore, accumulating evidence indicates that UHRF1 expression is closely associated with multiple clinicopathological parameters in cancer patients [17]. In colorectal cancer, elevated UHRF1 expression correlates with increased patient mortality. A similar trend is observed in bladder cancer, where UHRF1 expression is markedly upregulated in both superficial and invasive tumors relative to normal urothelial tissue, and its expression level positively correlates with tumor grade and aggressiveness [18]. Yang reported that UHRF1 overexpression serves as an independent prognostic factor for tumor recurrence in non-muscle-invasive bladder cancer [19], suggesting its utility as a molecular biomarker for predicting disease relapse. These findings are consistent with our experimental results, collectively supporting UHRF1 as a robust biomarker for tumor diagnosis and prognostic evaluation. However, the present study is among the first to characterize UHRF1 in the specific context of ATLL, where HTLV-1-driven epigenetic dysregulation is a hallmark of pathogenesis. UHRF1 functions as a key epigenetic regulator that maintains DNA methylation patterns and preserves silencing of tumor suppressor genes during replication [4,6,20–22]. Its aberrant overexpression in ATLL may therefore reflect, or contribute to, the epigenetic instability characteristic of HTLV-1-associated leukemogenesis, although the directionality of this relationship remains to be determined.

The molecular mechanisms underlying UHRF1 overexpression in the initiation and progression of ATLL remain incompletely understood. Functional cellular assays demonstrate that UHRF1 knockdown significantly suppresses proliferation and induces apoptosis in the HuT-102 ATLL and MT-4 ATLL cell line. UHRF1 silencing led to a substantial increase in early‑apoptotic cell populations in both cell lines, accompanied by elevated late‑apoptotic fractions in HuT‑102 cells, whereas necrotic cell proportions remained unchanged. These findings indicate that UHRF1 knockdown mainly affects apoptotic pathways instead of necrosis. Meanwhile, UHRF1 silencing produced divergent cell‑cycle phenotypes between the two cell lines: S‑phase accumulation was observed in HuT‑102 cells, whereas prominent G1‑phase arrest occurred in MT‑4 cells, suggesting that UHRF1 may be associated with altered cell cycle distribution in ATLL cell lines (HuT-102 cells and MT-4 cell). This result aligns with prior functional studies of UHRF1 in other tumor types, supporting its involvement in cell cycle regulation. Following UHRF1 knockdown in ATLL cells, we observed significant inhibition of cell growth and proliferation.

Previous studies have reported markedly reduced AMPK activity in renal and breast cancers, concomitant with aberrant UHRF1 overexpression in tumor tissues—indicating a robust clinical correlation between high UHRF1 expression and AMPK suppression [23,24]. Regarding the potential signaling pathways underlying these observations, we examined the relationship between UHRF1 and AMPK in ATLL. Immunoblotting confirmed that both total AMPK and phosphorylated AMPK (p-AMPK, Thr172) were detectable in HuT-102 cells. However, co-immunoprecipitation assays revealed no detectable stable interaction between UHRF1 and either AMPK or the PP2A catalytic subunit PPP2CA, suggesting that any functional crosstalk between UHRF1 and the AMPK axis is likely indirect. Moreover, UHRF1 knockdown did not significantly alter the expression of LKB1 or the PP2A regulatory subunit PPP2R4, nor the p-AMPK (Thr172)/AMPK ratio. Collectively, these findings indicate that a regulatory relationship between UHRF1 and AMPK-related proteins in ATLL could not be established under the tested conditions, and the molecular mechanisms underlying the pro-proliferative and anti-apoptotic effects of UHRF1 in ATLL remain to be elucidated.

In conclusion, our findings demonstrate that UHRF1 is markedly overexpressed in ATLL patient tissues and is significantly associated with poor clinical prognosis—establishing its potential as an independent prognostic biomarker. Functional knockdown experiments further provide preliminary evidence that UHRF1 is a critical driver of the malignant phenotype in ATLL cells. Nevertheless, this study has several limitations: First, it is a retrospective, single‑center analysis with a relatively small sample size (n = 72), and potential selection bias. Second, systematic profiling of immunophenotypic markers was not performed in our cohort; therefore, prospective studies incorporating comprehensive immunophenotypic characterization together with UHRF1 expression assessment are warranted for future investigations. Third, the proposed link between UHRF1 and AMPK-related signaling remains preliminary and correlative, lacking direct functional evidence (e.g., pharmacological rescue, phosphatase activity assays, or in vivo metabolic characterization). Fourth, due to limited sample size, the events‑per‑variable ratio may compromise the stability of hazard ratio estimates. Relatively short follow‑up also restricts our assessment of long‑term events and the durability of UHRF1‑associated prognostic effects. Thus, the independent prognostic value of UHRF1 needs validation in larger, multi‑center cohorts. Fifth, the densitometric analysis of western blots was based on three paired biological replicates; although no statistically significant differences were detected, densitometric confirmation across additional replicates remains warranted. Moreover, only single-direction Co-IP data are available; reciprocal Co-IP and supplementary binding assays are needed to further explore any potential interaction between UHRF1 and AMPK-pathway components, representing a direction for future work. In addition, functional validation was performed in two ATLL cell lines (HuT-102 and MT-4), and confirmation in additional ATLL models and primary cells is needed.

## Conclusion

This study establishes UHRF1 as an independent adverse prognostic indicator in ATLL, with high expression correlating with aggressive clinicopathological features and poor survival outcomes. Preliminary in vitro observations suggest a potential functional role in ATLL cell proliferation and apoptosis, though the underlying molecular mechanisms remain to be fully elucidated. Future studies should prioritize multi-center prospective validation of UHRF1 as a prognostic biomarker, expansion of in vitro analyses to additional ATLL cell lines and primary cells, and in vivo xenograft modeling to assess the biological and therapeutic relevance of UHRF1 in this disease.

## Supporting information

S1 Table Sensitivity analysis of multivariate Cox proportional‑hazard regression for predictors of PFS. (DOCX)

S2 Table Sensitivity analysis of multivariate Cox proportional‑hazard regression for predictors of OS. (DOCX)

S1 Raw Images Uncropped and unadjusted original blot images underlying Figs 3, 6, 8 and 9, including the loading controls and the overexposed LKB1/PPP2R4 blot. (PDF)
