# Supplementary material for: Elevated UHRF1 predicts unfavorable clinical outcomes in adult T-cell leukemia/lymphoma: Clinicopathological analysis and preliminary in vitro observations
Source: PLoS One. 2026 Nov 11;21(11):e0362674. doi: 10.1371/journal.pone.0362674 (PMC13754231; doi:10.1371/journal.pone.0362674)

Figure 3B_GAPDH

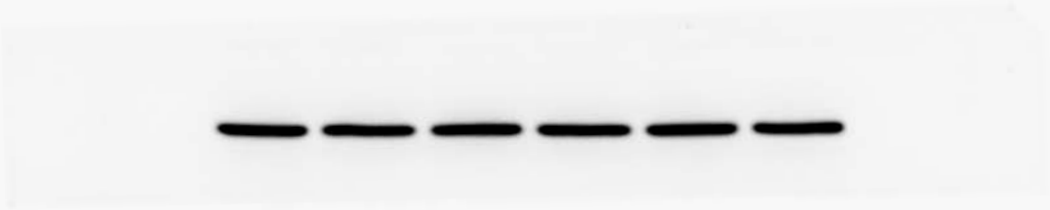

Figure 3B_UHRF1

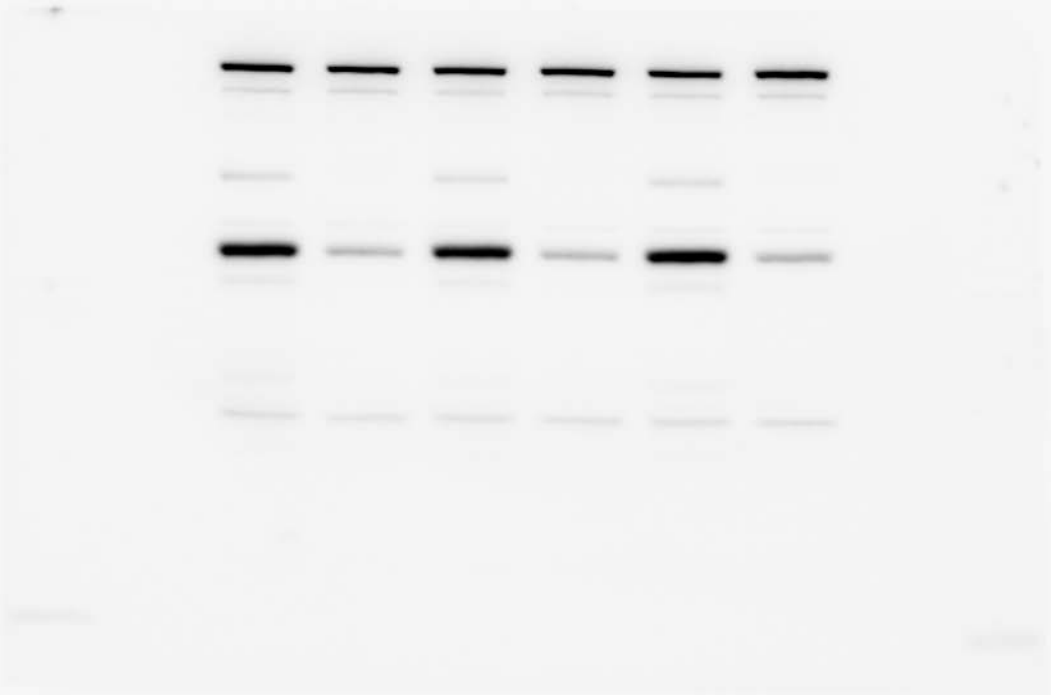

Figure 3D_actin

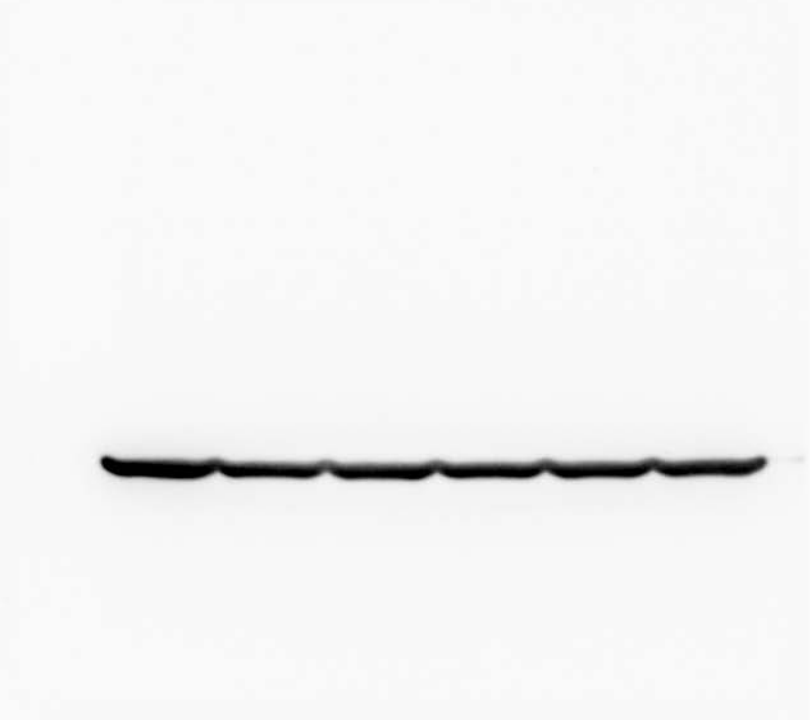

Figure 3D_UHRF1

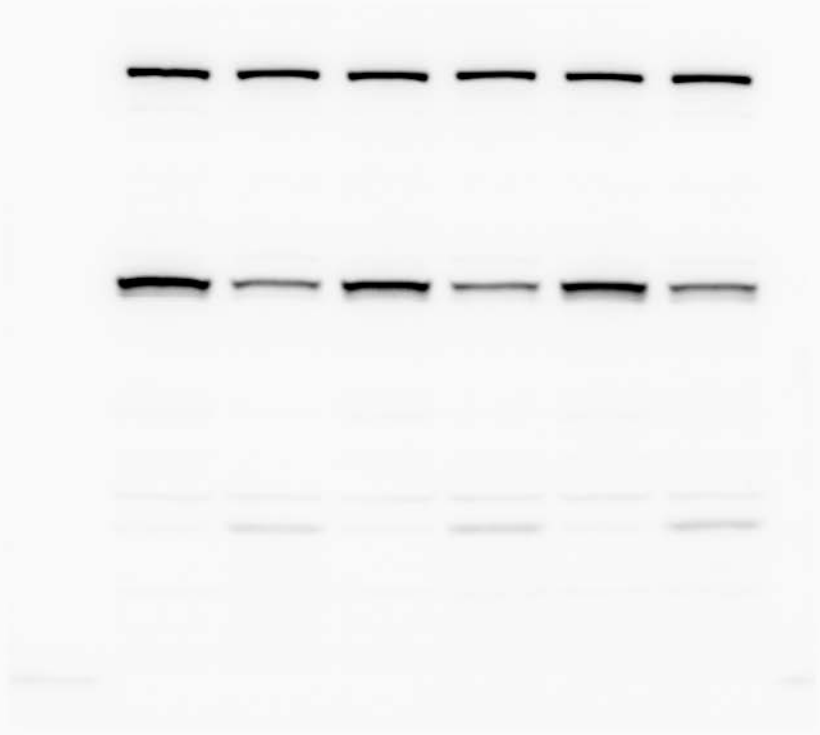

Figure 6_actin merge

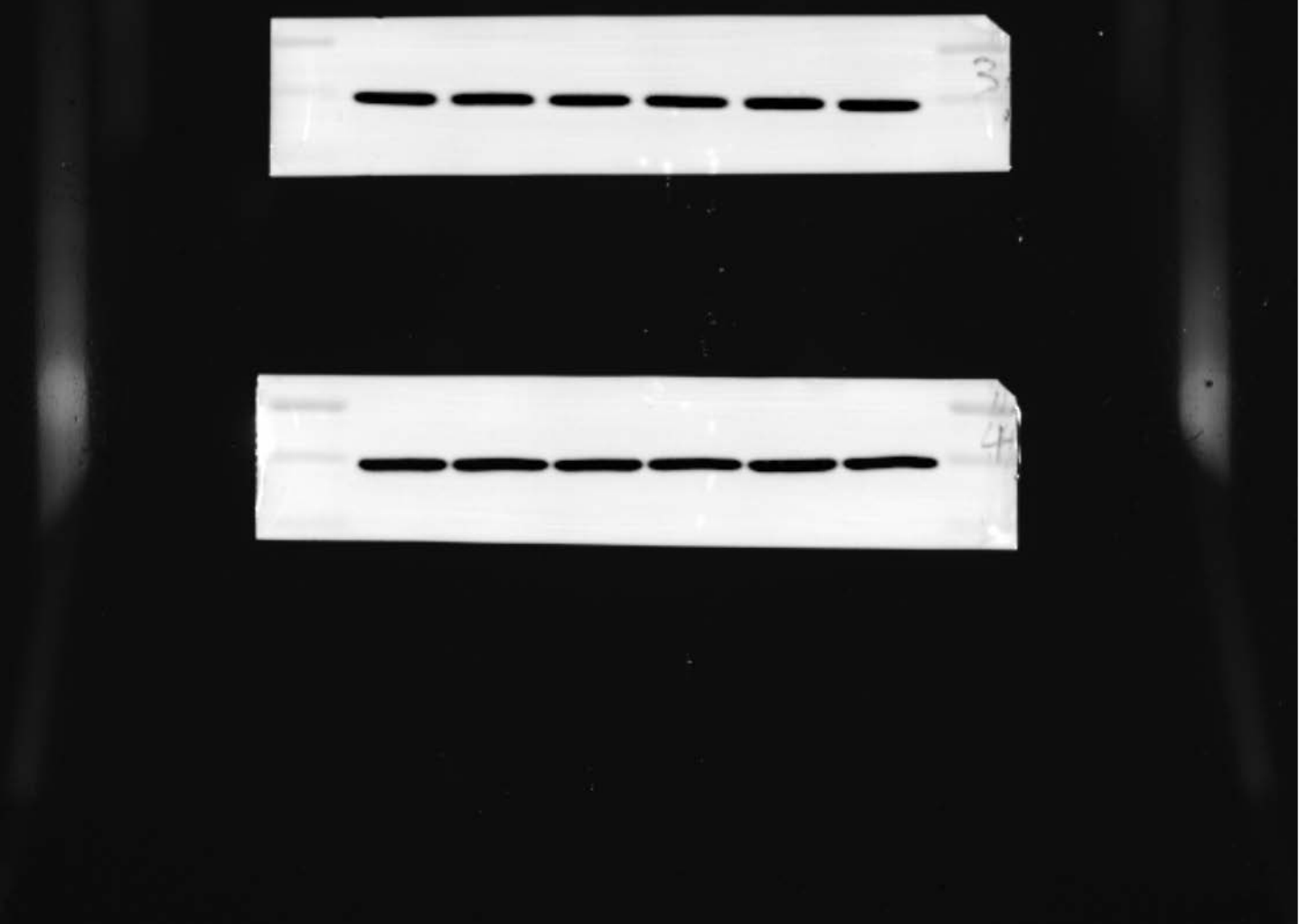

Figure 6_AMPK merge

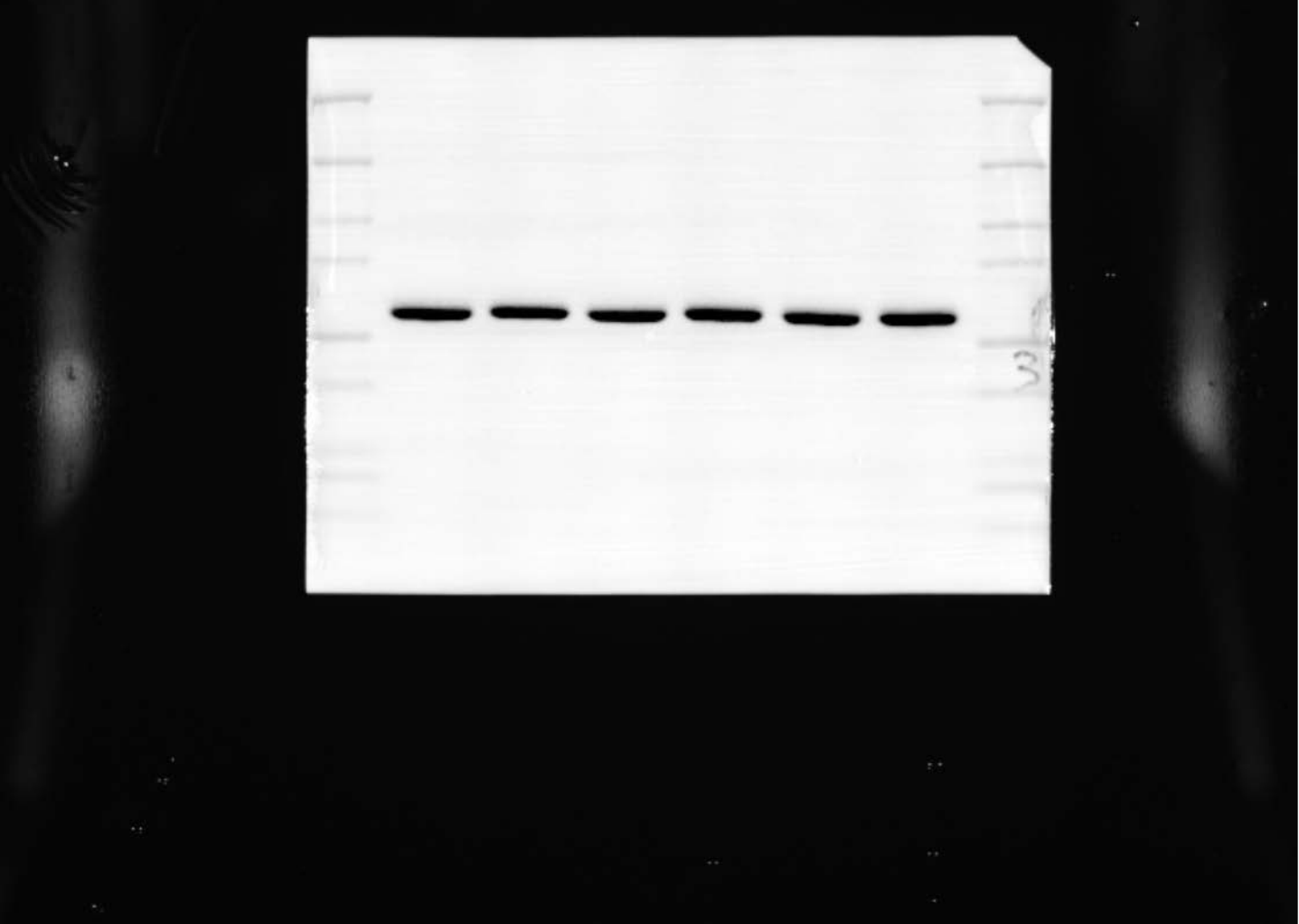

Figure 6_P-AMPK merge

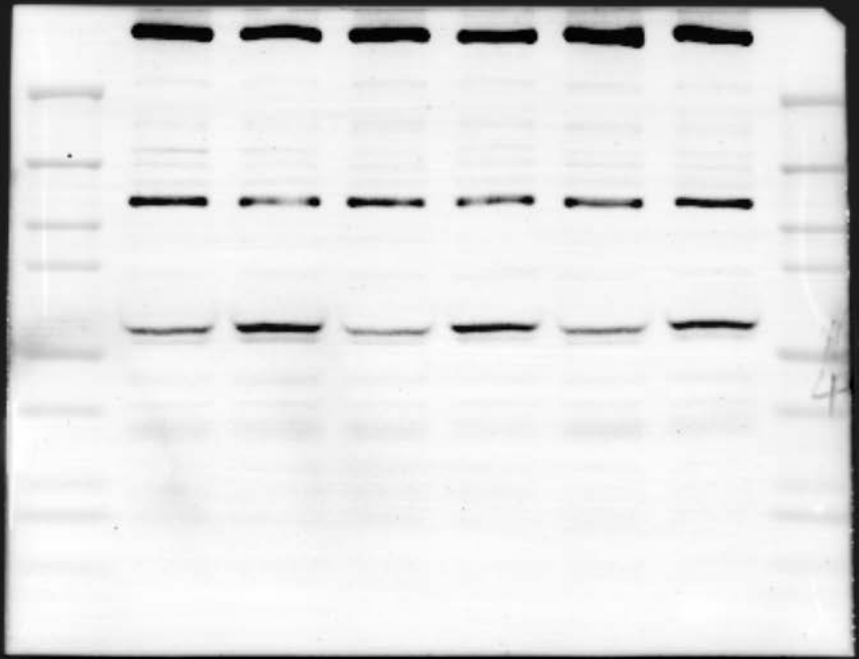

Figure 8A_AMPK

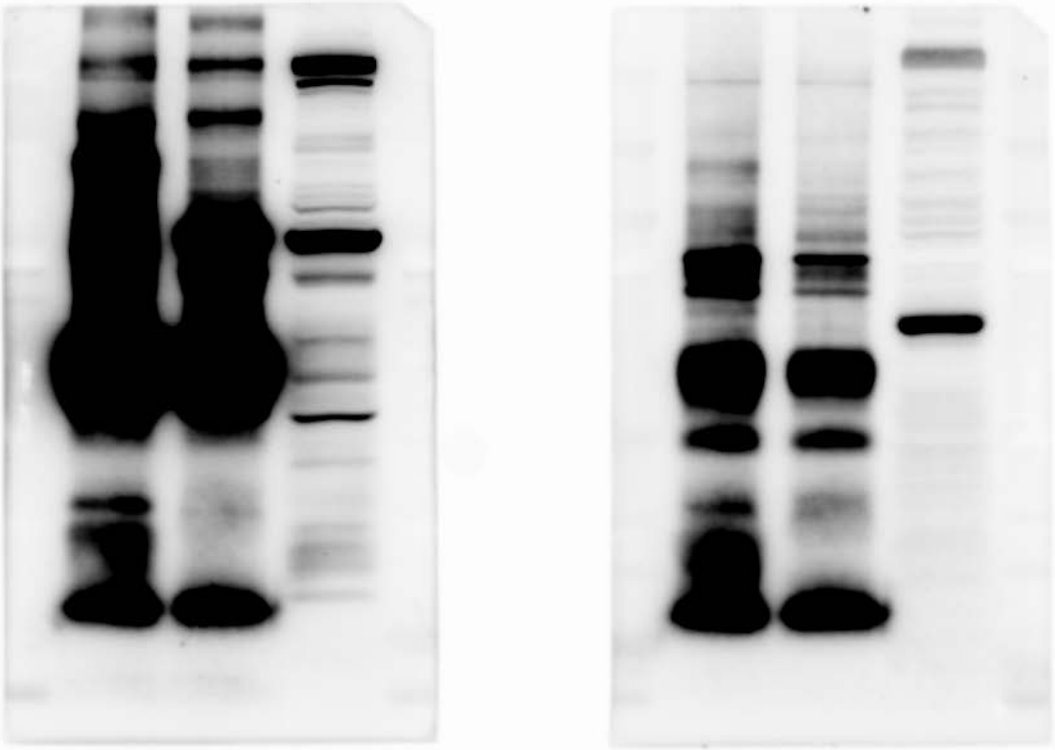

Figure 8A_UHRF1

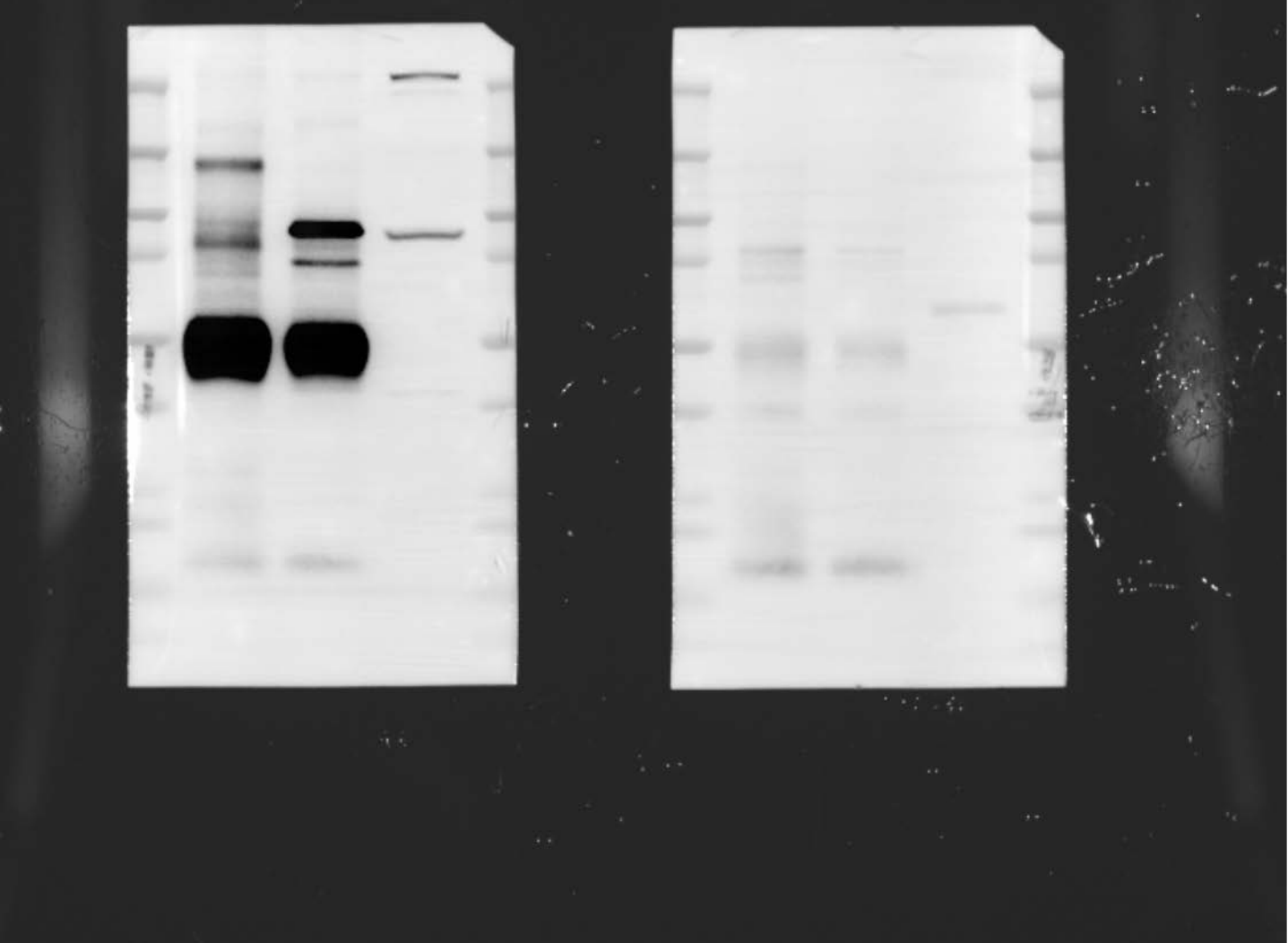

Figure 8B_PPP2CA

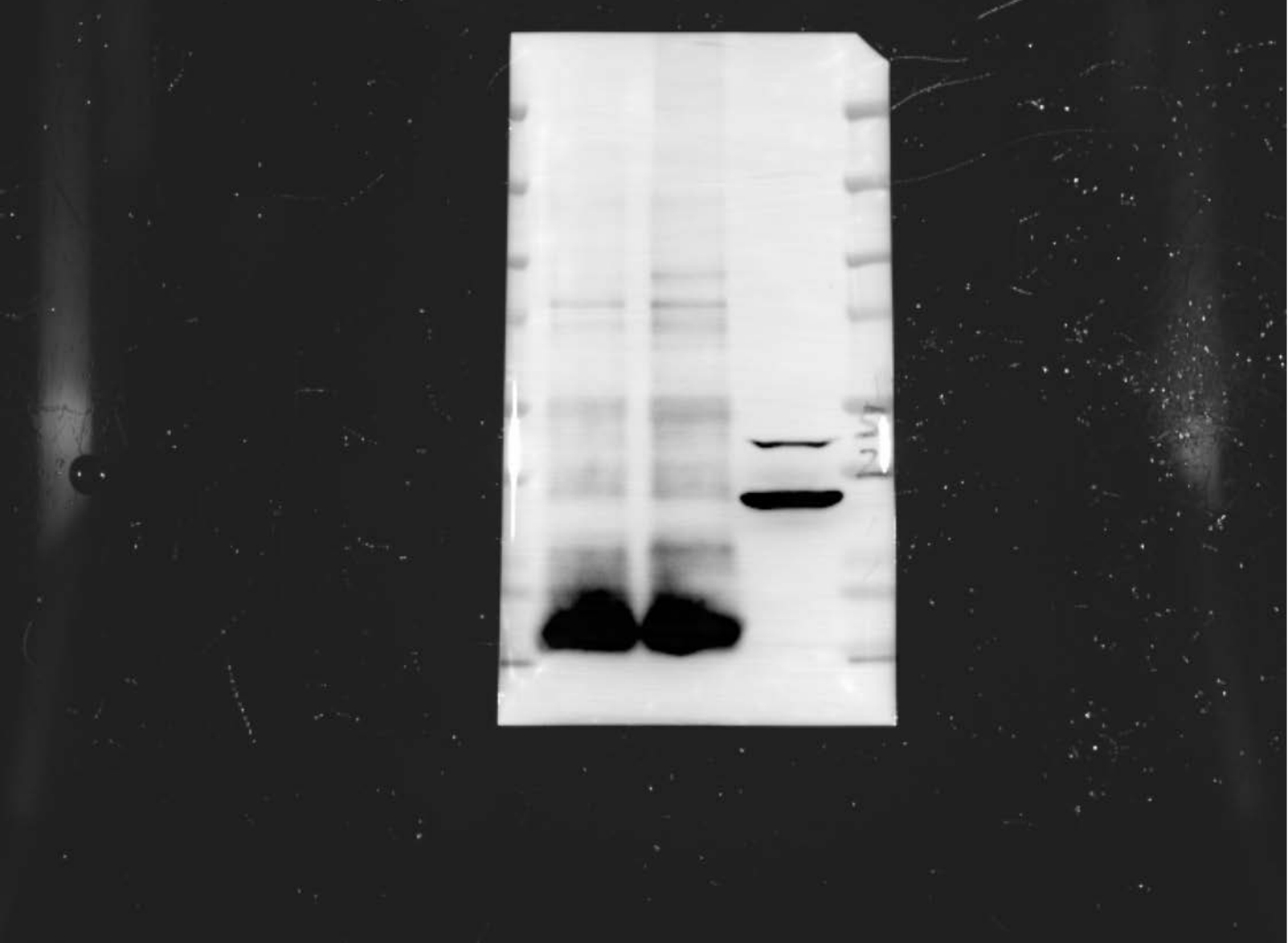

Figure 8B_UHRF1

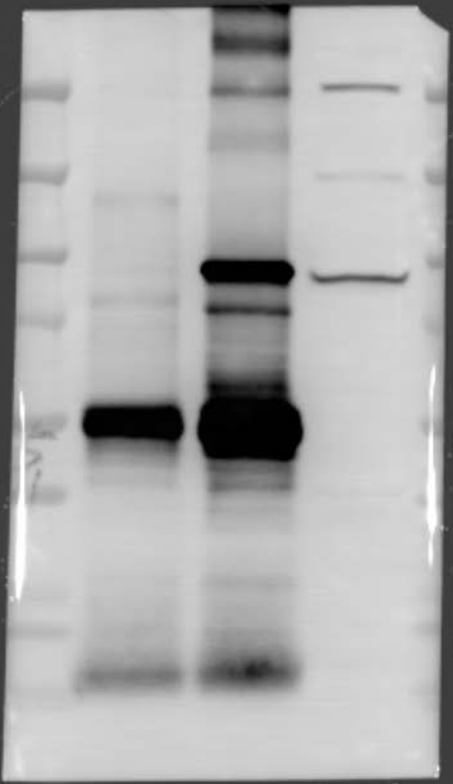

Figure 9_GAPDH

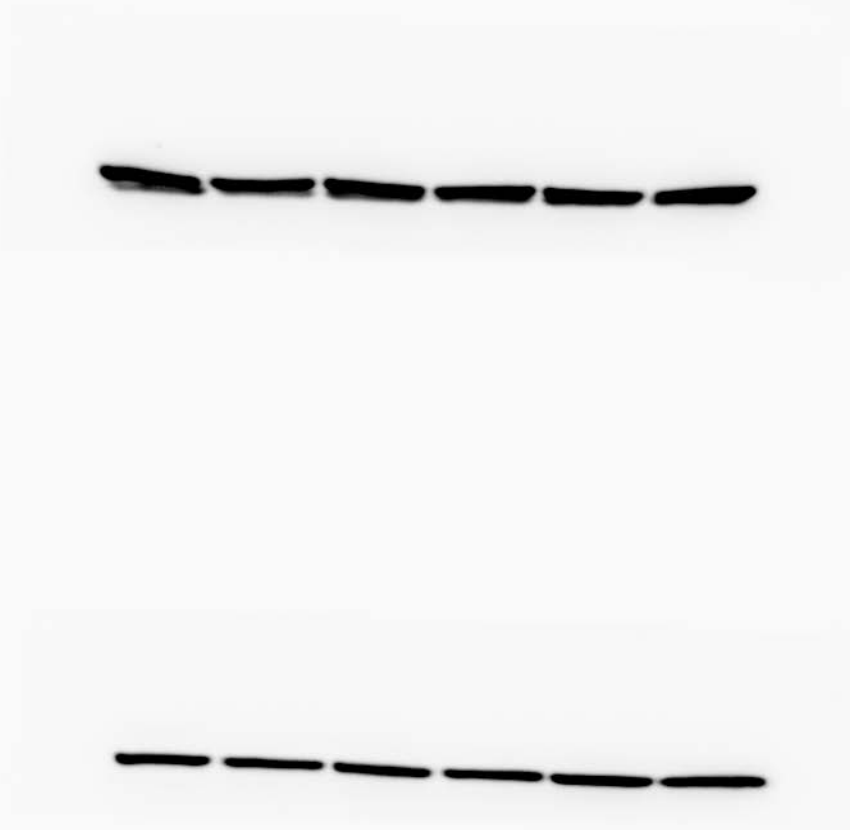

Figure 9_LKB1&PPP2R4

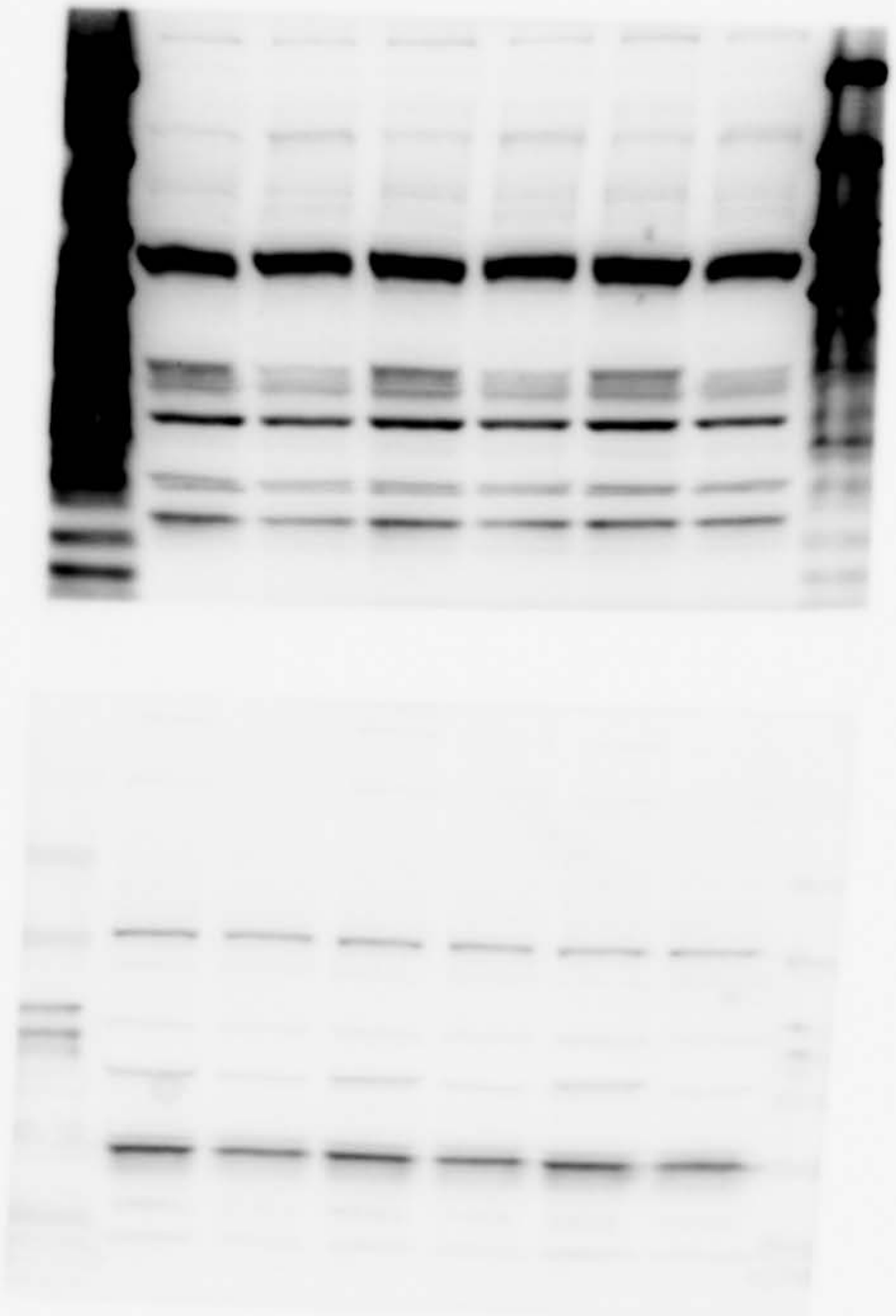

Figure 9_LKB1&PPP2R4_Overexposed

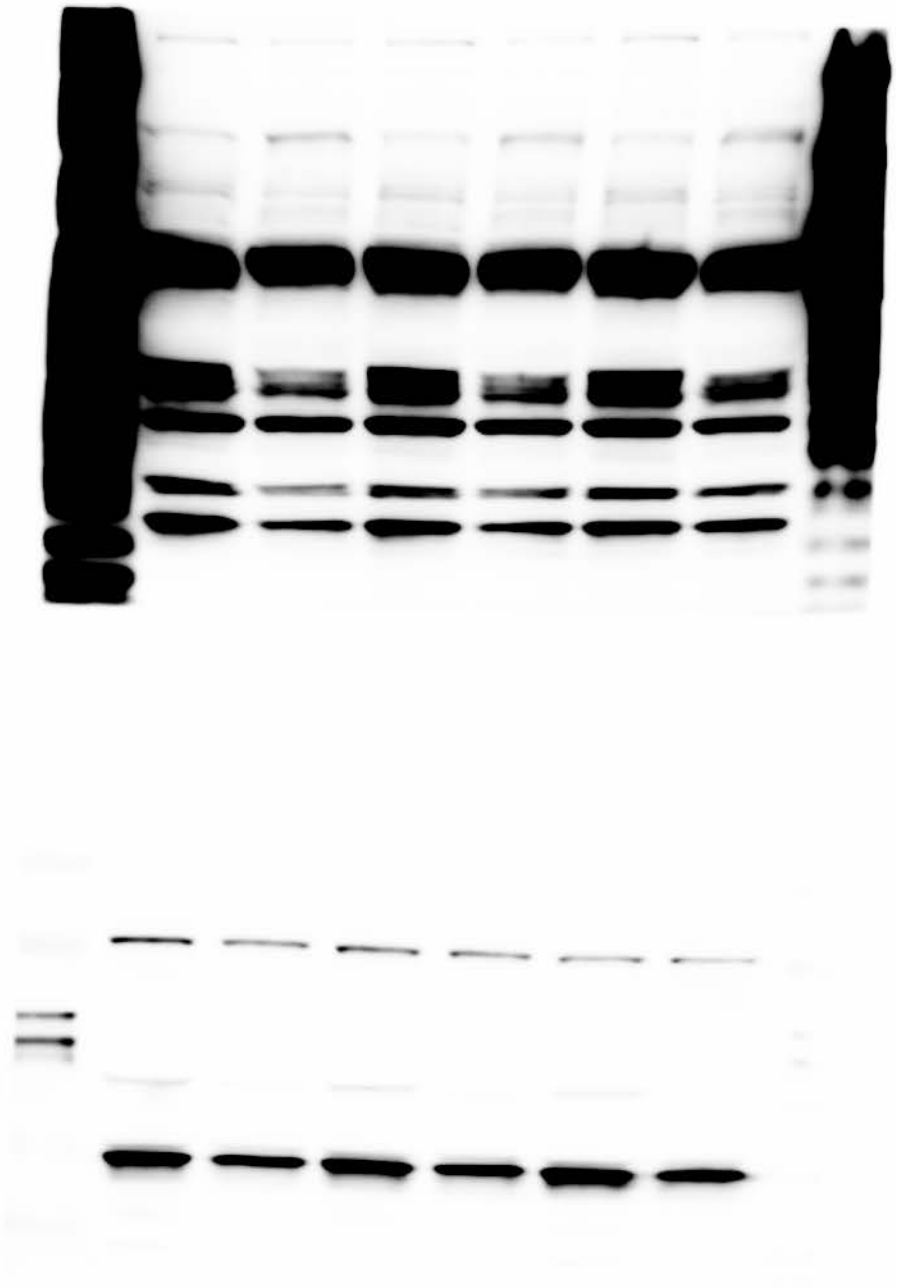

Supplement: Supplementary file 1 [file pone.0362674.s003.pdf]
