# Supplementary material for: Elevated UHRF1 predicts unfavorable clinical outcomes in adult T-cell leukemia/lymphoma: Clinicopathological analysis and preliminary in vitro observations
Source: PLoS One. 2026 Nov 11;21(11):e0362674. doi: 10.1371/journal.pone.0362674 (PMC13754231; doi:10.1371/journal.pone.0362674)
Supplement: Supplementary file 2 [file pone.0362674.s001.docx]

S1 Table. Sensitivity analysis of multivariate Cox proportional‑hazard regression for predictors of PFS

| Items | Multivariate Cox’s proportional hazard regression model | | | |
| --- | --- | --- | --- | --- |
|  | *P* value | HR | 95%CI | |
|  |  |  | Lower | Higher |
| ATL type (acute vs. lymphoma/chronic/smoldering) | 0.209 | 0.678 | 0.369 | 1.244 |
| B symptoms (yes vs. no) | 0.163 | 3.039 | 0.637 | 14.500 |
| Bone marrow invasion (yes vs. no) | 0.902 | 1.133 | 0.157 | 8.179 |
| β2-MG | 0.063 | 1.246 | 0.988 | 1.572 |
| UHRF1 protein expression (high vs. low) | **0.044** | 1.866 | 1.015 | 3.428 |
