# Supplementary material for: Elevated UHRF1 predicts unfavorable clinical outcomes in adult T-cell leukemia/lymphoma: Clinicopathological analysis and preliminary in vitro observations
Source: PLoS One. 2026 Nov 11;21(11):e0362674. doi: 10.1371/journal.pone.0362674 (PMC13754231; doi:10.1371/journal.pone.0362674)
Supplement: Supplementary file 3 [file pone.0362674.s002.docx]

S2 Table. Sensitivity analysis of multivariate Cox proportional‑hazard regression for predictors of OS

| Items | Multivariate Cox’s proportional hazard regression model | | | |
| --- | --- | --- | --- | --- |
|  | *P* value | HR | 95%CI | |
|  |  |  | Lower | Higher |
| ATL type (acute vs. lymphoma/chronic/smoldering) | **0.049** | 0.561 | 0.316 | 0.997 |
| B symptoms (yes vs. no) | 0.067 | 4.124 | 0.908 | 18.738 |
| Bone marrow invasion (yes vs. no) | 0.737 | 1.401 | 0.196 | 9.984 |
| UHRF1 protein expression (high vs. low) | **0.040** | 1.862 | 1.028 | 3.373 |
